# Effects of Berberine on Insulin-Sensitive Tissues: Nutrient-Derived Modulator of Metabolic Disease

**DOI:** 10.3390/cells15211984

**Published:** 2026-10-30

**Authors:** Maddox Harrison, Nicolaos Achilleos, Evangelia Tsiani

**Affiliations:** 1 Department of Health Sciences, Faculty of Applied Health Sciences, Brock University, St. Catharines, ON L2S 3A1, Canada; mh21kq@brocku.ca (M.H.); za25my@brocku.ca (N.A.); 2 Centre for Bone and Muscle Health, Applied Health Sciences, Brock University, St. Catharines, ON L2S 3A1, Canada

**Keywords:** berberine, insulin resistance, skeletal muscle, adipose tissue, hepatocytes

## Abstract

Insulin-sensitive tissues, including skeletal muscle, adipose tissue, and liver, are central regulators of systemic glucose and lipid homeostasis. Insulin signaling disruption in these tissues is a key pathological feature underlying insulin resistance and contributes to the development of metabolic disorders such as type 2 diabetes mellitus (T2DM), obesity, and liver disease. Increasing evidence suggests that berberine (BBR), a naturally occurring isoquinoline alkaloid derived from *Berberis* species, attenuates insulin resistance and exhibits antidiabetic, lipid-lowering, and anti-inflammatory properties across multiple metabolic tissues. This review analyzes current in vitro, in vivo, and clinical data examining the effects of BBR in insulin-sensitive tissues. In skeletal muscle, BBR promotes glucose consumption while in adipose tissue, it attenuates adipogenesis, lipid accumulation and inflammation. In hepatic tissue, BBR suppresses gluconeogenesis, improves lipid metabolism and reduces hepatic steatosis. Clinical trials report reductions in fasting glucose, serum lipids, visceral adiposity, and hepatic fat content. Collectively, current evidence indicates that BBR exerts multi-tissue insulin-sensitizing effects with therapeutic potential for metabolic disease. However, mechanistic human data directly linking tissue-specific actions remain limited. Future clinical studies incorporating tissue-specific metabolic assessments, dose–response analyses, metabolomic profiling, and consideration of sex- and age-related variability are warranted to fully elucidate its clinical efficacy.

## 1. Introduction

### 1.1. The Role of Insulin in Metabolic Homeostasis

Insulin is an anabolic peptide hormone secreted by pancreatic β-cells that regulates glucose, lipid, and protein metabolism [1,2]. In insulin-sensitive tissues, insulin binds the insulin receptor and promotes insulin receptor substrate (IRS) phosphorylation, activating the PI3K/Akt signaling pathway and leading to GLUT4 translocation and glucose uptake in skeletal muscle and adipose tissue, while promoting glycogenesis and suppressing gluconeogenesis in hepatocytes [1,2,3,4]. Insulin further regulates adipose tissue remodeling by influencing adipogenesis, lipid turnover, and adipokine secretion, thereby contributing to systemic metabolic homeostasis [2,4].

### 1.2. Impact of Insulin Resistance in Insulin-Sensitive Tissues

Insulin resistance is a worldwide metabolic health concern and a central feature in the pathogenesis of T2DM [5,6,7,8]. Its clinical importance stems from its role as an early and persistent driver of broader metabolic disease risk [5,6,7]. In insulin resistance, insulin-sensitive tissues, including skeletal muscle, adipose tissue, and the liver, exhibit impaired responsiveness to normal physiological concentrations of insulin. This reduced insulin action triggers compensatory hyperinsulinemia and contributes to disturbances in glucose uptake, lipid metabolism, and overall metabolic homeostasis [2,5,6,7]. Impaired insulin receptor, IRS-PI3K-Akt, and downstream signaling reduce glucose uptake, increase hepatic glucose output, and alter adipokine secretion, contributing to obesity, dyslipidemia, metabolic syndrome, and T2DM [2,3,4,7]. In 2022, approximately 830 million individuals were living with diabetes, a substantial increase from fewer than 200 million in 1990 [8].

Skeletal muscle plays a central role in whole-body glucose homeostasis, accounting for approximately 70–80% of insulin-stimulated glucose disposal. Consequently, skeletal muscle insulin resistance significantly impairs GLUT4 translocation and glucose uptake, contributing to systemic glucose dysregulation [2,7]. Hepatic insulin resistance increases gluconeogenesis and contributes to hepatic steatosis, fasting hyperglycemia, Non-Alcoholic Fatty Liver Disease (NAFLD), and Metabolic Dysfunction-Associated Fatty Liver Disease (MAFLD) [2,9,10]. In adipose tissue, insulin resistance disrupts lipolytic control and adipokine homeostasis, increasing non-esterified fatty acid release and promoting ectopic lipid deposition in liver and muscle [2,4,11]. Together, dysfunction of skeletal muscle, adipose tissue, and liver creates a network of metabolic dysregulation that drives T2DM, obesity, NAFLD, and MAFLD [2,4,7,9,10,11].

### 1.3. The Role of Phytochemicals in Regulation of Insulin Action

Plant-derived bioactive molecules have gained attention for their ability to modulate insulin signaling and attenuate metabolic dysfunction in insulin-sensitive tissues [12,13,14]. Phytochemicals are of interest because their structural diversity and multi-target effects may complement conventional pharmacotherapy [12,13,14]. Metformin, the first-line treatment for T2DM, was originally derived from French lilac (Galega *officinalis*) and improves insulin action partly through AMPK activation [15]. AMPK, a cellular energy sensor, promotes GLUT4 translocation; its activation in insulin-sensitive tissues enhances insulin sensitivity and promotes glucose and lipid metabolic homeostasis [16,17,18].

In addition to metformin, other plant-derived compounds such as berberine (BBR), curcumin, resveratrol, and quercetin have been shown to improve insulin sensitivity and activate AMPK signaling [12,13,14,16,18]. Despite evidence supporting the potential insulin-sensitizing properties of these plant-derived compounds, most phytochemicals lack approved clinical applications due to limited evidence from clinical trials [12,14]. Further translational studies are needed to evaluate their safety, bioavailability, pharmacokinetics, and therapeutic efficacy in humans.

### 1.4. The Structure, Metabolism, and Bioavailability of Berberine

**Figure 1:**
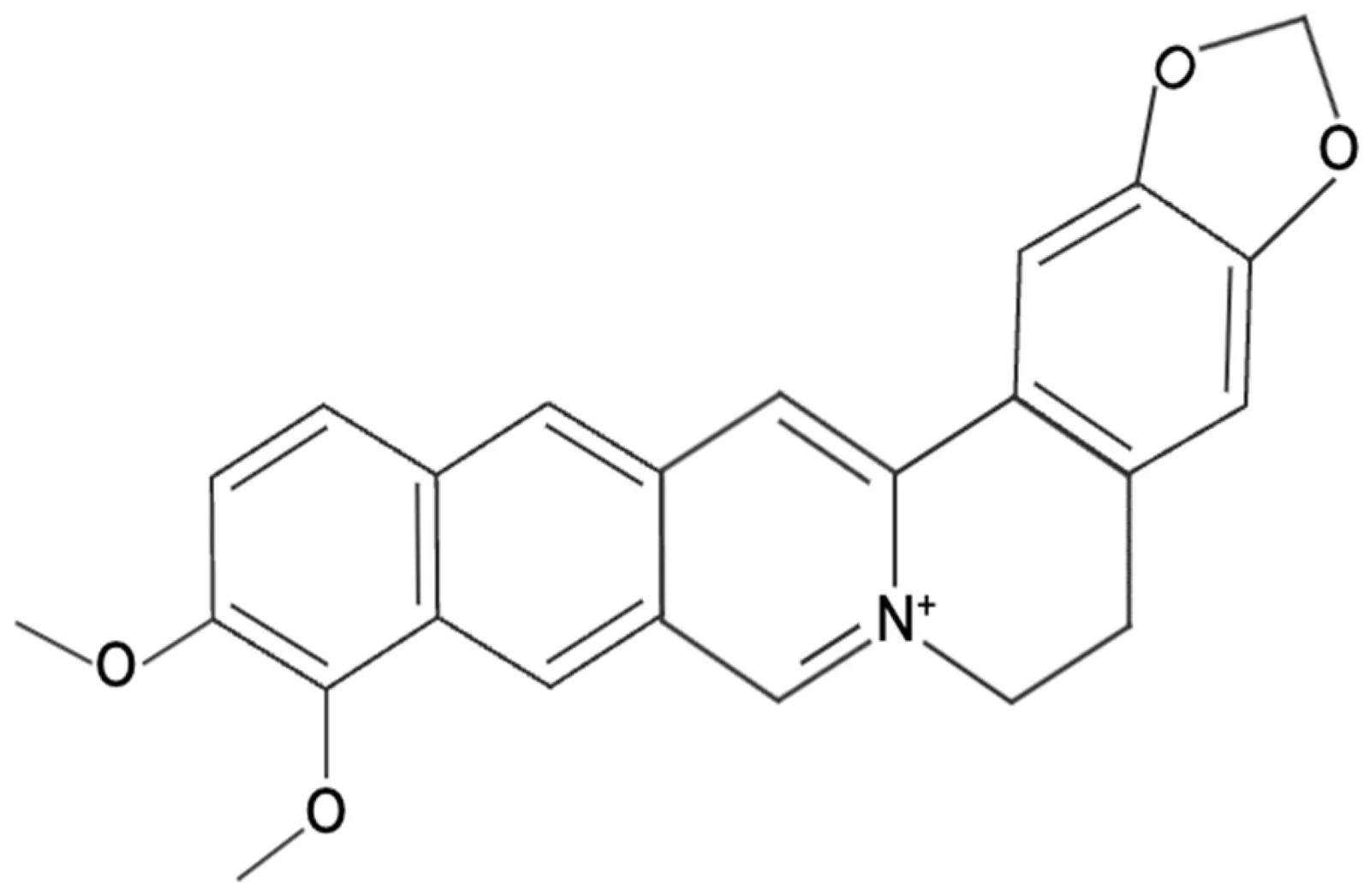
Chemical Structure of Berberine (BBR). Created in BioRender. Harrison, M. (2026) https://BioRender.com/pu6po5p.

BBR has gained considerable scientific attention for its broad spectrum of pharmacological activities. It is a naturally occurring isoquinoline alkaloid [19]. BBR(C20H18NO4+; Figure 1) possesses a planar quaternary ammonium structure composed of four fused rings and multiple methoxy groups, a configuration that imparts its characteristic yellow color and biological reactivity [19]. BBR is primarily isolated from the roots, rhizomes, and bark of plants including *Berberis vulgaris*, *Coptis chinensis*, and *Hydrastis canadensis* [19]. Orally administered BBR exhibits low bioavailability owing to poor intestinal absorption, extensive first-pass metabolism, and active efflux by P-glycoprotein (P-gp) transporters in enterocytes [19,20]. Once absorbed, BBRundergoes phase I and phase II metabolism primarily in the liver and intestines. Its major metabolites, berberrubine, demethyleneberberine, thalifendine, and jatrorrhizine, have been shown to retain pharmacological activity in animal studies and may contribute to BBR’s systemic effects despite its low plasma concentration [19,20,21].

Metabolic effects of BBR have been investigated previously, with the earliest English-language manuscript identified in our literature search published in 2002 and demonstrated reduced glucose output in BBR-treated HepG2 cells [22]. Subsequent studies and reviews further established its glucose- and lipid-lowering effects. Therefore, this review focuses on evidence published from January 2015 to August 2026 to emphasize more recent mechanistic and translational findings in skeletal muscle, adipose tissue, and liver. This evidence was gathered using databases such as PubMed and Google Scholar with the following keyword searches: “Berberine and muscle tissue and insulin”, “berberine and adipocytes and insulin”, “berberine and adipose and insulin”, “berberine and hepatocytes and insulin”, and “berberine and liver and insulin”. Studies were included if they evaluated BBR or BBR-based formulations in skeletal muscle, adipose tissue, liver/hepatocytes, animal models of metabolic dysfunction, or human metabolic disease. Studies that focus on cancer, neurological disease, infection, or non-metabolic outcomes were excluded. After searching each database, a total of 73 studies were included in the current review.

The majority of studies included in this review were peer-reviewed and underwent methodological validation and data-integrity assessment. Methodology sections of the original publications were assessed to confirm the validity of bioassay procedures, and reported statistical analyses were reviewed for appropriateness. Sample size was reported for each original human clinical trial in the results section of this review. Doses, concentrations, and treatment durations are reported using the units provided in the original studies to preserve study-level reporting. The terms “increase” or “decrease” in the summary tables denote statistically significant changes as reported in the source studies. Only significant outcomes were included. Readers seeking detailed statistical parameters such as confidence intervals, effect sizes, or study power should refer to the original publications. The present review uniquely provides a narrative synthesis of the effects of BBR organized by the major insulin-sensitive tissues, skeletal muscle, adipose tissue, and the liver. This structure addresses the potential of BBR to act as a treatment for metabolic disease and allows for a global understanding of BBR through its mechanisms in these tissues. By systematically reporting the specific cell types, animal models, treatment doses and durations, observed effects, and associated mechanisms, this review provides researchers and students with a comparative reference for evaluating differences across studies and identifying gaps that require further investigation. This structure highlights the extent to which BBR’s effects translate across preclinical and clinical evidence. Furthermore, we discussed translational outcomes and identified and addressed limitations such as bioavailability, metabolite exposure, and human outcomes.

## 2. Effects of Berberine on Insulin-Sensitive Cells: In Vitro Studies

### 2.1. Effects of Berberine on Skeletal Muscle Cells

In cytokine-treated C2C12 myoblasts, Poudel et al. [23] found that treatment with BBR reduced IL-1β- and TNF-α-induced NF-κB nuclear translocation, iNOS expression, and stress kinase signaling, including p38 MAPK, SAPK/JNK, MKK4, C-JUN, and SMAD4 Table 1. BBR also restored AMPK phosphorylation, increased SIRT1 and PGC-1α, and preserved mitochondrial electron transport chain complexes I, II, III, and V. These findings suggest that BBR protects skeletal muscle progenitor cells by activating the AMPK–SIRT1–PGC-1α pathway, suppressing inflammatory signaling and improving mitochondrial function [23].

In another study, exposure of insulin-resistant C2C12 to BBR increased glucose uptake, insulin-stimulated GLUT4 translocation, and phosphorylation of AKT. The data indicate that BBR improves skeletal muscle insulin signaling by limiting glucocorticoid receptor interference with the IRS1–PI3K–AKT–GLUT4 pathway [24].

In C2C12 myocytes [25], BBR increased mitochondrial number, AMPK phosphorylation, citrate synthase activity, and PGC-1α protein expression. BBR also upregulated genes involved in mitochondrial biogenesis, oxidative phosphorylation, and fatty acid oxidation, including *mtTFA*, *NRF1*, *COXIV*, *CytC*, *PDK4*, *CPT1β*, *MCAD*, and *LCAD*. Seahorse-based oxygen consumption analysis showed reduced basal respiration but increased maximal respiratory capacity, suggesting improved mitochondrial reserve function. These results suggest that BBR promotes skeletal muscle mitochondrial biogenesis and fatty acid oxidation through an AMPK–PGC-1α-dependent mechanism [25].

In L6 myotubes [26], BBR reduced fructose-induced IL-1β, IL-6, TNF-α, ROS, and MDA levels while restoring IL-10, SOD, CAT, and GSH levels. BBR also increased Nrf2, HO-1, p-AKT, GLUT4, 2-NBDG glucose uptake, and AMPK phosphorylation. Compound C attenuated the BBR-mediated AMPK activation, Nrf2/HO-1 expression, and glucose uptake, supporting AMPK involvement. BBR further increased AMP and the AMP/ATP ratio, reduced AMPD1, and increased ADSL, suggesting that BBR attenuates fructose-induced skeletal muscle insulin resistance partly through AMPD1/ADSL-mediated activation of AMPK and downstream Nrf2 and AKT/GLUT4 signaling [26].

In Palmitate (PA)-induced insulin-resistant C2C12 myotubes [27], BBR reduced *TNFα* and *IL-6* mRNA levels, lipid droplet accumulation, and the atrophy markers FBXO32 and MuRF-1. BBR also reduced Bax, increased Bcl2, lowered P62, increased LC3B, and restored FUNDC1 protein expression, suggesting reduced apoptosis and improved FUNDC1-related mitophagy. FUNDC1 knockdown weakened the anti-atrophy effect of BBR, indicating that BBR protects against PA-induced skeletal muscle atrophy partly through FUNDC1-mediated mitochondrial autophagy [27].

Overall, these studies indicate that BBR protects skeletal muscle cells by enhancing insulin signaling and glucose uptake, activating AMPK-dependent pathways, promoting mitochondrial biogenesis and fatty acid oxidation, and reducing inflammation, oxidative stress, and muscle atrophy.

### 2.2. Effects of Berberine on Adipocytes

Dong et al. [28] treated 3T3-L1 adipocytes and preadipocytes with BBR, and a significant reduction in differentiation was observed in preadipocytes, which was associated with reduced lipid content Table 2. A decrease in ROS (reactive oxygen species) was also observed in BBR-treated 3T3-L1 adipocytes, as well as a decrease in leptin and resistin protein levels. However, the expression of the adipogenic and antioxidant GPx was markedly increased in BBR-treated 3T3-L1 adipocytes [28].

In 3T3-L1 adipocytes [29], BBR reduced intracellular triglyceride content and increased p-HSL (Ser565), *ATGL* mRNA, ATGL, and p-AMPK levels, without significantly altering GPAT3 expression. Pretreatment with Compound C attenuated the BBR-mediated increases in ATGL and p-HSL. These findings indicate that BBR lowers triglyceride accumulation in mature adipocytes partly through AMPK-associated upregulation of ATGL [29].

Mature 3T3-L1 adipocytes [30] treated with BBR had significantly reduced *IL-6*, *TNF-α*, and *leptin* mRNA levels. BBR also increased glucose transport and Visfatin expression, while adiponectin remained unchanged. These findings demonstrate that BBR improves adipocyte glucose metabolism and suppresses inflammatory cytokine expression [30].

Lin et al. [31] treated 3T3-L1 preadipocytes with BBR and found upregulated beige adipocyte markers, PRDM16 and UCP1, and reduced white adipocyte marker resistin. BBR also enhanced basal and maximal oxygen consumption rates and ATP production. BBR reduced miR-92a expression and increased RBM4a. Overexpression of miR-92a or depletion of RBM4a abolished BBR-induced thermogenic gene expression and mitochondrial activity. These findings show that BBR promotes beige adipogenesis in 3T3-L1 cells through a mechanism involving miR-92a–RBM4a signaling [31].

Primary mouse preadipocytes isolated from epididymal WAT and treated with BBR [32], had reduced preadipocyte differentiation and proliferation, intracellular triglyceride content, lipid accumulation, and adipogenic markers, including PPARγ, C/EBPα, and aP2. BBR also decreased *Gal-3* mRNA and protein expression, while Gal-3 overexpression abolished the BBR-mediated suppression of adipocyte differentiation and proliferation. BBR reduced Gal-3 promoter activity, destabilized Gal-3 mRNA, and increased let-7d expression, suggesting that BBR inhibits adipogenesis partly through let-7d-mediated downregulation of Gal-3 [32].

In insulin-resistant 3T3-L1 adipocytes [33], BBR increased glucose usage and adiponectin secretion. A reduction in fatty deposits and a significant increase in HIF3A, IRS-1, and GLUT4 protein levels were seen with BBR treatment [33].

Yang et al. [34] used differentiated porcine adipocytes and found increased glycerol release and decreased intracellular triglyceride content and lipid droplet accumulation with BBR treatment. BBR increased p-AMPKα and p-ATGL, decreased *Perilipin A* mRNA and protein expression, and upregulated fatty acid oxidation-related markers, including PGC-1α, TFAM, CPT-1, and UCP2. Compound C and AMPKα1 knockdown attenuated these effects, indicating AMPK dependence [34].

Shan et al. [35] found increased SIRT1 expression, insulin-stimulated glucose uptake, and phosphorylation of LKB1, AMPK, ACC, and AKT in 3T3-L1 adipocytes exposed to BBR. In TNF-α-treated adipocytes, BBR improved insulin-stimulated glucose uptake and p-AKT (Ser473), while SIRT1 inhibition weakened these effects. These findings suggest that BBR improves adipocyte glucose metabolism through SIRT1-dependent activation of the LKB1–AMPK pathway and restoration of insulin signaling [35].

In another study, in differentiated 3T3-L1 white adipocytes and HIB1b brown adipocytes [36], BBR increased mitochondrial number, SIRT1 and UCP1 expression, thermogenic and fatty acid oxidation gene expression, and the NAD+/NADH ratio while reducing lipid droplet size and PPARγ acetylation. AICAR produced similar effects, whereas Compound C and AMPK knockdown reduced BBR-mediated NAD+/NADH changes, UCP1 expression, and PPARγ deacetylation. SIRT1 knockdown also blocked BBR-induced UCP1 expression and lipid reduction, suggesting that BBR promotes adipocyte remodeling and thermogenesis through AMPK-dependent activation of SIRT1 and PPARγ deacetylation [36].

Li et al. [37] treated primary porcine intramuscular preadipocytes with BBR during adipogenic differentiation and observed a dose-dependent reduction in intracellular triglyceride accumulation. BBR significantly decreased adipocyte differentiation and suppressed *PPARγ* and *adiponectin* mRNA expression, indicating attenuation of adipogenic gene programs. These findings show that BBR inhibits adipogenic differentiation through a multi-gene regulatory pattern involving repression of key adipogenic genes [37].

Li et al. [38] used TNF-α-treated 3T3-L1 adipocytes and found that treatment with BBR increased SIRT3 expression and reduced TNF-α-induced inflammatory signaling, including p-IKKα/β, p-IκBα, p-p65, p-JNK, p-p38, and p-ERK. BBR also decreased iNOS and COX-2 expression, while the SIRT3 inhibitor AGK7 diminished these anti-inflammatory effects. These results suggest that BBR suppresses TNF-α-induced adipocyte inflammation through SIRT3-dependent inhibition of NF-κB and MAPK signaling [38].

Zhang et al. [39] treated differentiating 3T3-L1 adipocytes with BBR, evodiamine, or both compounds (BBE). BBE treatment significantly reduced intracellular lipid droplet accumulation and lowered triglyceride content more effectively than either compound alone. BBE also upregulated Prdm16 and Cidea mRNA, and increased UCP1 and PGC-1α protein expression, indicating enhanced browning and thermogenic programming [39].

Overall, in vitro evidence indicates that BBR enhances insulin signaling and glucose utilization in adipocytes, activates AMPK, suppresses adipogenesis, reduces proinflammatory cytokine expression, and modulates adipokine-related signaling.

### 2.3. Effects of Berberine on Hepatocytes

Treatment of insulin-resistant HepG2 cells [40] with BBR resulted in significantly increased 2-NBDG glucose uptake, reduced AChE activity, upregulated α7-nAChR protein expression, and reduced inflammatory signaling, IL-6 secretion, IKKβ Ser181 phosphorylation, and NF-κB p65 expression Table 3. These findings indicate that BBR attenuates insulin resistance and suppresses inflammatory signaling in hepatocytes through activation of the cholinergic anti-inflammatory pathway [40].

Wei et al. [41] exposed HepG2 cells to palmitate to induce gluconeogenic and lipogenic dysregulation, and found that treatment with BBR significantly reduced G6Pase, PEPCK, SREBP-1, FAS, and ACCα, and raised the p-ACCα/ACCα ratio, indicating suppressed gluconeogenesis and lipogenesis with enhanced fatty-acid oxidation. BBR also reduced HNF-4α and miR-122 levels, both of which were elevated by palmitate. These findings show that BBR attenuates palmitate-induced hepatic gluconeogenesis and lipogenesis through suppression of the HNF-4α–miR-122 pathway [41].

Treatment of MIHA and HepG2 cells with BBR [42] resulted in increased *LDLR* mRNA levels and miR-373 expression. BBR increased EGR1 expression, and EGR1 knockdown attenuated BBR-induced miR-373 upregulation. Increased miR-373 levels were associated with reduced *AKT1* mRNA and protein expression, and decreased mTOR, RICTOR, and p-S6K levels. These findings suggest that BBR may suppress hepatic steatosis-associated AKT–mTOR–S6K signaling through EGR1-mediated induction of miR-373, although the study did not directly measure lipid accumulation or insulin sensitivity [42].

Treatment of primary mouse hepatocytes with BBR [43] increased glucose uptake, lactate output, intracellular AMP levels, p-AMPK, and plasma membrane GLUT1 expression, while reducing SIRT3 expression, oxygen consumption rate, and hepatic glucose production. BBR also decreased intracellular cAMP levels and suppressed glucagon-induced gluconeogenesis by promoting PEPCK1 ubiquitination/degradation. Although AMP accumulation activated AMPK and promoted glucose uptake, the inhibition of glucagon signaling and PEPCK1 stabilization was reported to be independent of AMPK activation. These results suggest that BBR improves hepatic glucose handling by inhibiting SIRT3, shifting hepatocyte metabolism toward AMPK-associated glucose uptake while suppressing glucagon-driven gluconeogenesis [43].

In high-glucose-induced insulin-resistant AML12 hepatocytes, BBR increased glucokinase (GK) levels and intracellular glycogen content, suggesting enhanced hepatic glucose storage. These findings indicate that BBR may improve hepatocyte glucose metabolism by upregulating GK and promoting glycogen synthesis under high-glucose conditions [44].

Zhu et al. [45] induced insulin resistance in HepG2 cells using 25 mM glucose and 200 µM palmitic acid, and found significantly reduced SCD1 protein levels with BBR treatment. Total hepatic triglyceride content was increased by glucose and palmitic acid treatment, while BBR attenuated this response. BBR treatment also significantly increased p-AMPK protein levels, suggesting that BBR regulates SCD1 through the AMPK–SREBP-1c pathway [45].

In oleic acid-treated HepG2 cells [46], BBR reduced lipid accumulation, decreased FAS and ChREBP expression, increased glucose consumption, and suppressed gluconeogenesis; these effects were blocked by AMPKα1 knockout. These results demonstrate that BBR improves hepatic glucose and lipid metabolism through AMPKα1-dependent signaling [46].

In insulin-resistant HepG2 cells [47], BBR increased glucose uptake while reducing inflammatory mediators, including TNF-α, IL-1β, IL-6, and IL-8, and increasing IL-10. BBR upregulated PPM1B, PPARγ, LRP1, GLUT4, IRS1, IRS2, PI3K, and AKT, while reducing NF-κB p65, JNK, and the p-IKKβ (Ser181)/IKKβ ratio. PPM1B knockdown worsened inflammatory signaling and glucose metabolic dysfunction, and BBR partially reversed these effects, suggesting that BBR improves insulin-resistant HepG2 cell function partly through cAMP/PKA/PPM1B-mediated suppression of inflammatory signaling and restoration of the IRS–PI3K–Akt pathway [47].

Yu et al. [48] treated primary mouse hepatocytes with oleic acid to induce steatosis and found that treatment with BBR significantly reduced lipid droplet accumulation and lowered intracellular triglyceride content. BBR also decreased mitochondrial oxygen consumption, reduced both basal and maximal respiration, lowered ATP production, and increased the phosphorylation of AMPK and ACC. These findings indicate that BBR alleviates hepatocellular lipid accumulation by reducing ATP production and increasing AMPK and ACC phosphorylation [48].

In TNF-α -induced insulin resistant primary mouse hepatocytes [49], BBR suppressed p-MEKK1, p-MEK1/2, and p-ERK1/2, reduced p-IRS1 (Ser612), and restored insulin-stimulated IRS1 tyrosine phosphorylation and p-AKT expression. These findings suggest that BBR attenuates TNF-α-induced hepatic insulin resistance by targeting MEKK1/MEK signaling, thereby limiting ERK1/2-mediated inhibitory IRS1 phosphorylation and restoring AKT-dependent insulin signaling [49].

Xu et al. [50] used palmitate (PA)-treated HepG2 cells and found that treatment with BBR increased SIRT1 and Opa1 protein expression, restored mitochondrial membrane potential, increased ATP content, and improved mitochondrial morphology. BBR also increased insulin-stimulated p-AKT and glucose consumption, indicating improved hepatocyte insulin signaling and glucose metabolism. These effects were attenuated by SIRT1 silencing, suggesting that BBR attenuates PA-induced hepatic insulin resistance partly by activating SIRT1/Opa1 signaling and preserving mitochondrial architecture [50].

In cholesterol-overloaded primary mouse hepatocytes [51], BBR improved cell viability and reduced cholesterol-induced apoptosis, ROS generation, 4-HNE levels, NADPH oxidase activity, and iNOS expression. BBR decreased Keap1 expression while increasing Nrf2 and HO-1 expression, indicating activation of the antioxidant Keap1–Nrf2/HO-1 pathway. Keap1 upregulation or Nrf2 knockdown weakened the anti-apoptotic effects of BBR, suggesting that BBR protects cholesterol-overloaded hepatocytes primarily by reducing oxidative stress through Nrf2/HO-1 signaling [51].

BBR reduced basal and glucagon-stimulated glucose production while downregulating the gluconeogenic enzymes G6Pase and PEPCK in primary rat hepatocytes [52]. BBR increased NO and cGMP levels, enhanced PKG signaling, increased p-AKT1, and reduced p-CREB expression. AKT1 silencing or pathway inhibition weakened BBR-mediated suppression of gluconeogenesis, suggesting that BBR inhibits hepatic glucose production through AKT1/MAPK-dependent activation of NO/cGMP/PKG signaling and downstream repression of CREB-mediated G6Pase and PEPCK expression [52].

BBR increased glucose uptake and reduced glucosamine-induced ROS and H_2_O_2_ accumulation in HepG2 cells [53]. BBR also restored *Bmal1* and *Clock* mRNA and protein levels, suggesting correction of glucosamine-induced circadian disruption. Bmal1 knockdown reduced Clock expression and blocked the BBR-mediated improvement in glucose uptake, indicating that BBR improves glucose metabolism and redox homeostasis partly through Bmal1/Clock-dependent signaling [53].

Chen et al. [54] used HepG2 cells and found that BBR increased *FGF21* mRNA and protein levels PPARγ and GLUT2 expression were also increased, while PPARγ knockdown attenuated the BBR-induced FGF21 and GLUT2 upregulation. In glucosamine-treated insulin-resistant HepG2 cells, BBR restored p-AKT and p-GSK3β levels, and this effect was weakened by FGF21 knockdown. These findings suggest that BBR improves hepatocyte insulin signaling partly through PPARγ-mediated induction of FGF21 and downstream GLUT2-associated glucose-handling pathways [54].

In HepG2 and BRL-3A, [55BBR attenuated the fructose-induced IL-6, TNF-α, ROS, and MDA levels while restoring IL-10, SOD activity, and GSH levels. BBR also increased Nrf2, HO-1, p-AMPK, AMP, the AMP/ATP ratio, and *ADK* mRNA and protein levels. ADK knockdown abolished the ability of BBR to restore p-AMPK, AMP levels, antioxidant defenses, and inflammatory markers, suggesting that BBR protects hepatocytes from fructose-induced oxidative and inflammatory injury partly through ADK-mediated AMPK/Nrf2 activation [55].

Yang et al. [56] used OA-treated HepG2 cells and found that treatment with BBR reduced TG content, lipid accumulation, *AKR1B10* mRNA and protein levels, and AKR1B10 enzymatic activity while increasing glucose uptake and glucose consumption., BBR increased PPARα-related markers, including CPT-1 and GK, while reducing PPARγ and downstream metabolic proteins, including ACC1, CD36, GLUT2, PEPCK, and G6Pase. BBR also increased the p-GSK3β/GSK3β ratio, suggesting improved glycogen synthesis-related signaling. AKR1B10 knockdown or inhibition attenuated the effects of BBR, indicating that BBR attenuates OA-induced hepatic lipid and glucose metabolic dysfunction partly through AKR1B10-dependent regulation of PPAR signaling [56].

Zhang et al. [57] used FFA-induced insulin-resistant HepG2 cells and found that treatment with BBR increased glucose consumption and 2-NBDG glucose uptake, while upregulating *PPARα* and *PPARγ* mRNA levels. BBR also reduced the FFA-induced increase in *GLUT1* mRNA levels. The use of PPARα/PPARγ inhibitors weakened BBR-mediated improvements in glucose uptake, suggesting that BBR attenuates FFA-induced hepatic insulin resistance partly through PPAR-dependent mechanisms [57].

Treatment of PA-induced insulin-resistant HepG2 cells with BBR reduced intracellular triglyceride and total cholesterol levels and decreased lipid droplet accumulation in PA-treated HepG2 cells. BBR activated PI3K/Akt signaling while suppressing the STING pathway, and AKT inhibition weakened the lipid-lowering effects of BBR. These results indicate that BBR protects hepatocytes from PA-induced lipid accumulation partly by activating PI3K/Akt signaling and inhibiting STING-mediated inflammatory/metabolic dysfunction [58].

BBR or metformin alone attenuated the fat (OA/PA)-induced intracellular lipid accumulation in HepG2 cells, with the combination resulting in the greatest effect. Combined treatment increased p-AMPK/AMPK while suppressing SREBP1 and FASN expression. Compound C abolished the reduction in lipid accumulation and triglyceride content, decreased p-AMPK, and restored SREBP1 and FASN levels, supporting AMPK-dependent inhibition of lipogenesis. These findings suggest that BBR enhances metformin-mediated protection against OA/PA-induced hepatocyte lipid accumulation through activation of AMPK and downstream suppression of SREBP1/FASN signaling [59].

**Figure 2:**
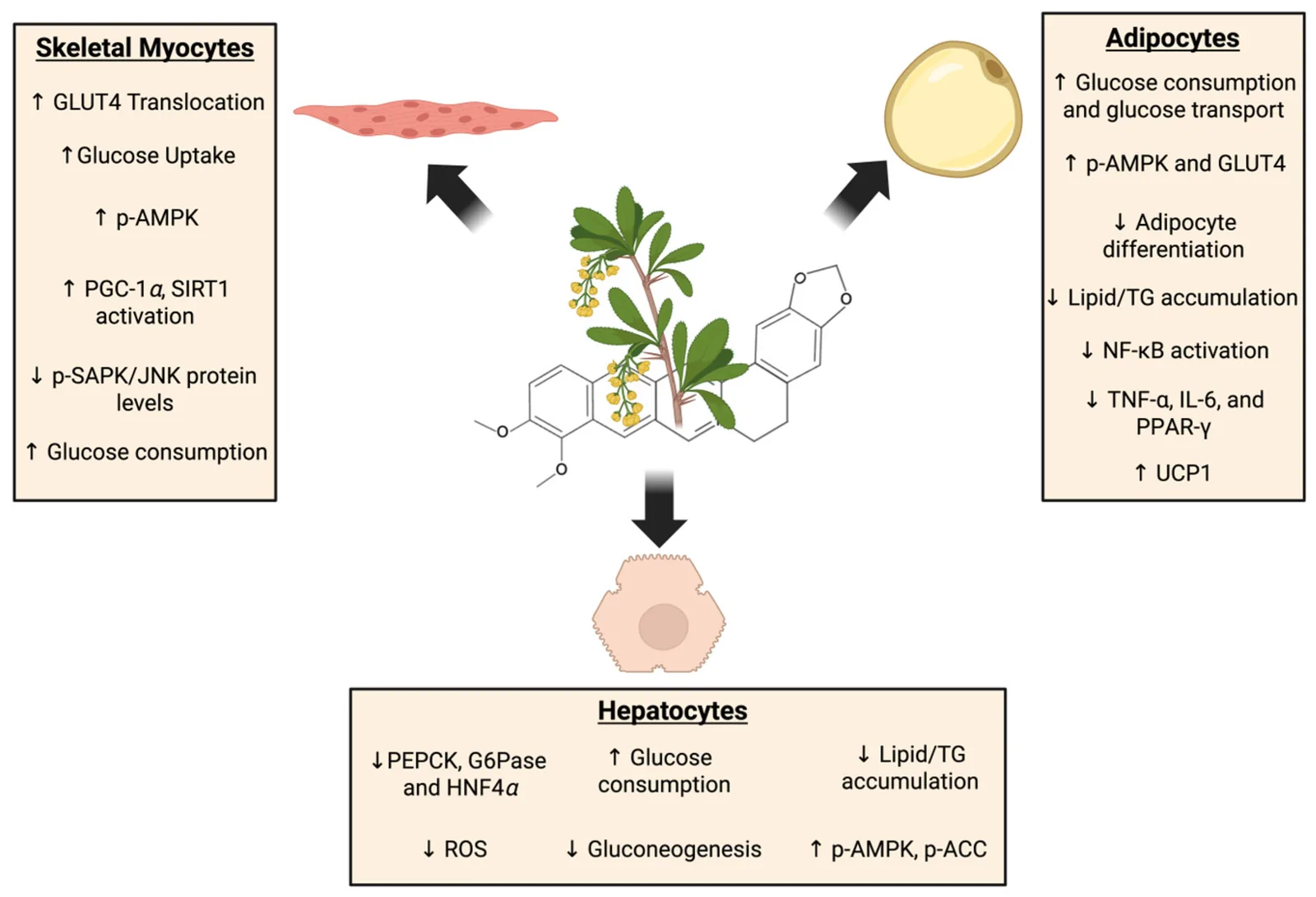
Effects of Berberine on Insulin-Sensitive Cells, Including Skeletal Myocytes, Adipocytes, and Hepatocytes. Created in BioRender. Harrison, M. (2026) https://BioRender.com/yi1yoys.

**Figure 3:**
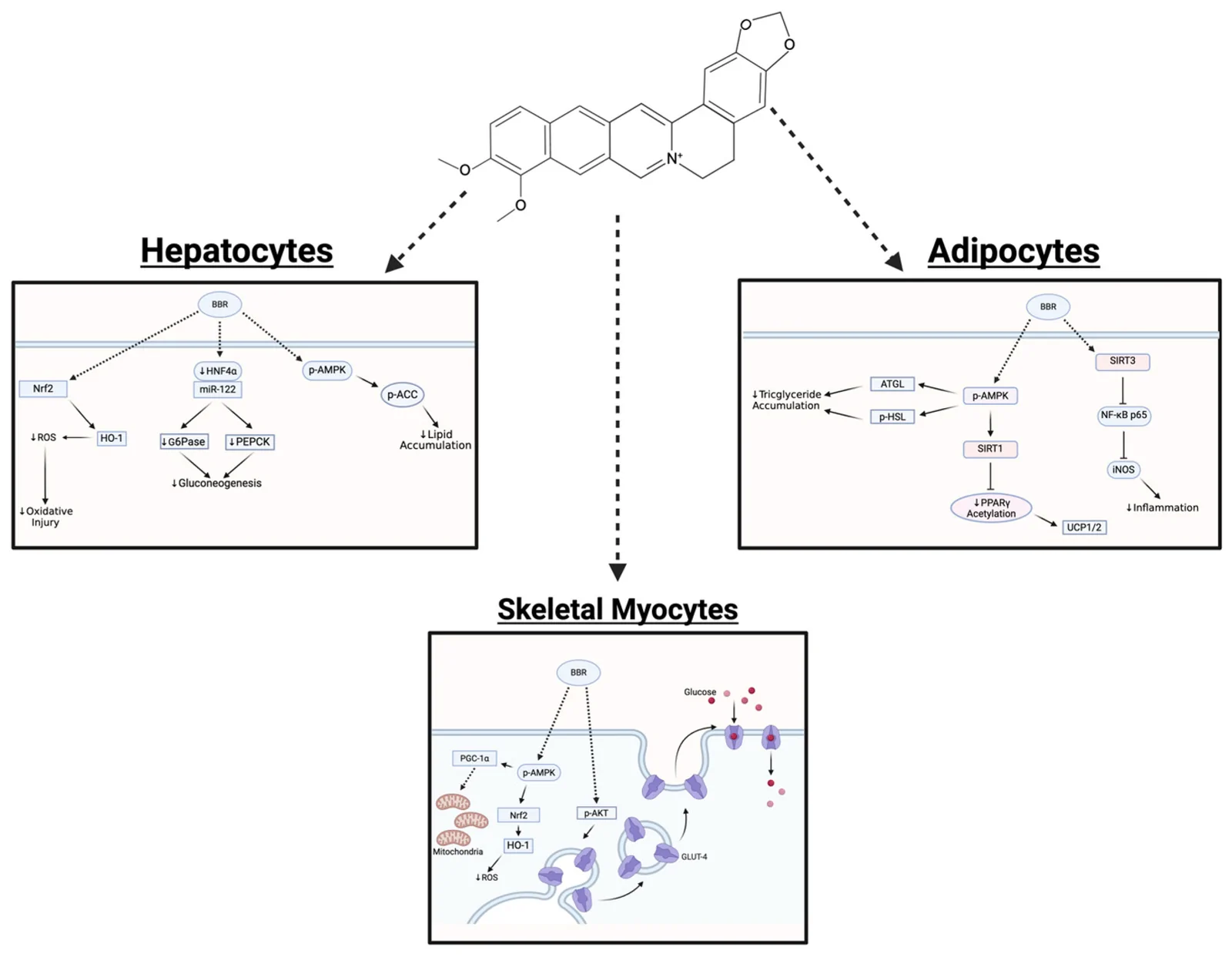
Potential Mechanisms of Berberine on Insulin-Sensitive Cells, Including Skeletal Myocytes, Adipocytes, and Hepatocytes. Created in BioRender. Harrison, M. (2026) https://BioRender.com/hp9mu7k.

Overall, the evidence outlined in the above sections indicates that BBR attenuates insulin resistance and increases glucose uptake in skeletal muscle cells through pathways involving AMPK, GLUT4, AKT, and mitochondrial regulation. In adipocytes, BBR inhibits differentiation and proliferation while reducing pro-inflammatory signaling and lipid deposition. In hepatocytes, BBR reduces gluconeogenesis and lipid accumulation while increasing glucose consumption through AMPK and PI3K/AKT-related pathways Figure 2 and Figure 3.

## 3. Effects of Berberine on Insulin-Sensitive Tissue: Preclinical Evidence

### 3.1. Effects of Berberine on Control Chow-Fed Animals

Yu et al. [60] evaluated whether BBR could attenuate aging-related cognitive decline, insulin resistance, and skeletal muscle dysfunction in Wistar rats Table 4. Morris water maze testing showed that BBR improved learning and memory performance in aged rats, reducing escape latency and increasing platform-crossing frequency and target-quadrant path length. BBR also reduced fasting insulin, fasting glucose, and triglyceride levels, lowered IPGTT AUC, and improved insulin-stimulated GLUT4 translocation in aged skeletal muscle. In muscle tissue, BBR restored muscle fiber number, increased ATP production, reduced ROS levels, and upregulated p-AMPK, SIRT1, and PGC-1α. These findings suggest that BBR protects against aging-related insulin resistance and skeletal muscle dysfunction partly through improved mitochondrial function and activation of the AMPK–SIRT1–PGC-1α pathway [60].

### 3.2. Effects of Berberine on Animals: High Fat Diet Model

In high glucose/high fat diet-induced diabetic hamsters [61], BBR reduced body weight, liver weight, hepatic fat accumulation, blood glucose, insulin, total cholesterol, triglycerides, free fatty acids, LDL-C, ApoB, MDA, TBARS, and 8-isoprostane levels, while improving glucose tolerance and increasing SOD activity Table 5. BBR also increased skeletal muscle *GLUT4* mRNA and hepatic *LDLR* mRNA expression, suggesting improved peripheral glucose handling and hepatic cholesterol clearance. These findings indicate that BBR improves hyperglycemia, dyslipidemia, hepatic lipid accumulation, and oxidative stress in diabetic hamsters [61].

In another study, the effects of BBR in male Sprague–Dawley rats with high-fat diet-induced non-alcoholic fatty liver disease (NAFLD) were investigated [62]. BBR significantly reduced hepatic triglyceride accumulation and serum ALT and AST levels, indicating attenuation of hepatic steatosis and liver injury. BBR also increased the mRNA and protein expression of carnitine palmitoyltransferase-1α (CPT-1α), microsomal triglyceride transfer protein (MTTP), and glucokinase (GCK), suggesting enhanced mitochondrial fatty acid β-oxidation, improved hepatic triglyceride export, and increased hepatic glucose utilization. Histological analyses further demonstrated reduced hepatic lipid accumulation compared with untreated high-fat diet controls [62].

In HFD-fed male C57BL/6J mice [63], receiving BBR improved glucose tolerance and insulin sensitivity without altering body weight or food intake. BBR reduced hepatic steatosis, liver p-JNK1, and hepatic *IL-1β* and *TNFα* mRNA levels, while also decreasing lipogenic genes including *ACC*, *FAS*, and *SREBP1c*. In adipose tissue, BBR decreased p-JNK1, IL-1β, TNFα, and the percentage of M1 macrophages, while total macrophage infiltration and M2 macrophage percentage were not significantly altered. These findings demonstrate that BBR attenuates hepatic steatosis and adipose-liver inflammation primarily through anti-inflammatory effects [63].

Sun et al. [64] administered BBR to HFD-fed male C57BL/6J mice and found that BBR reduced serum and liver triglyceride levels. *Fas*, a gene involved in fatty acid synthesis, was upregulated during HFD feeding but significantly reduced following BBR treatment, while *Cyp4a10*, a gene involved in fatty acid oxidation, was increased following BBR treatment [64].

In HFD-fed male C57BL/6 mice [65], BBR improved insulin sensitivity and glucose tolerance, reduced fasting serum insulin, and lowered epididymal fat mass relative to body mass. In adipose tissue, BBR decreased adipocyte size, CD11c+ macrophage infiltration, CD11c expression in SVF cells, caspase-1 activation, and IL-1β release from epididymal adipose explants. Adoptive-transfer experiments in macrophage-depleted ob/ob mice further showed that BBR improved glucose and insulin tolerance, while the autophagy inhibitor 3-MA reversed this effect. These findings suggest that BBR attenuates insulin resistance partly by inducing AMPK-dependent macrophage autophagy, thereby suppressing NLRP3 inflammasome activation in adipose tissue macrophages [65].

In male Wistar rats that were fed an HFD to induce insulin resistance [66], improved fasting blood glucose levels and HOMA-IR were observed after BBR administration, while body weight and visceral fat mass remained unaffected. BBR also reduced triglycerides and LDL-C and prevented HFD-induced hepatic weight gain. HFD feeding decreased *Bifidobacterium* and increased *Escherichia coli* levels and led to LPS plasma elevations, all of which were reversed by BBR. Furthermore, BBR decreased pro-inflammatory hepatic markers (TLR4 and TNF-α) and increased *IR* and *IRS-1* mRNA levels [66].

Wang et al. [67] examined whether BBR could reduce adipose tissue fibrosis in HFD-fed male C57BL/6J mice. BBR attenuated systemic insulin resistance, improved glucose intolerance, and reduced fasting insulin, plasma free fatty acids, body weight, and fat accumulation, and decreased *collagen type I*, *III*, and *VI* mRNA expression in adipose tissue. BBR also increased AMPK and ACC phosphorylation while reducing TGF-β1, macrophage abundance, and M1 inflammatory markers TNFα and iNOS. The increase in M2 markers Arg1 and Ym1 suggests that BBR attenuates adipose fibrosis partly through AMPK activation and reduced macrophage-mediated inflammation [67].

Li et al. [68] showed that BBR administration in male ICR mice significantly reduced fasting blood glucose and endogenous hepatic glucose production. These reductions were accompanied by decreased *PEPCK* and *G-6-Pase* mRNA and protein expression, indicating suppression of hepatic gluconeogenesis. BBR also decreased hepatic triacylglycerol accumulation and non-esterified fatty acid levels, which were associated with reduced expression of Facd and Kct, key enzymes in β-oxidation. BBR reduced hepatic lipid accumulation by suppressing SIRT3 activity, leading to reduced mitochondrial pyruvate carrier 1 (MPC1) expression through preservation of its inactive acetylated status while HFD diet favored acetylated MPC1 expression and increased hepatic lipid accumulation [68].

In HFD-induced obese male C57BL/6 mice [69], BBR administration attenuated body weight gain and fasting hyperglycemia and improved glucose tolerance and insulin sensitivity. In eWAT, BBR decreased adipocyte size, collagen deposition, collagen I, fibronectin, α-SMA, and PDGFR-α expression. BBR also suppressed HIF-1α expression and reduced LOX mRNA, suggesting that BBR alleviates obesity-associated adipose fibrosis partly by inhibiting HIF-1α–LOX-mediated extracellular matrix remodeling [69].

In HFD-fed male C57BL/6J mice, BBR reduced fat content/body mass ratio, serum leptin, hyperinsulinemia, hepatic lipid accumulation, and liver size while improving glucose tolerance and insulin sensitivity. BBR also increased UCP1, TH, p-STAT3, and oxygen consumption, suggesting enhanced thermogenic energy expenditure. In WAT, BBR reduced IL-6, IL-1β, TNF-α, MCP1/CCL2, and CD11b+F4/80+ macrophage recruitment while increasing IL-10 and M2 macrophage markers, including ARG1, MRC1, CHIL3, and Rentla. Reduced caspase-3, caspase-8, Bax, and IRE1α in M2 macrophages further suggest that BBR preserves anti-inflammatory macrophage polarization by limiting M2 macrophage apoptosis and ER stress [70].

In HFD-fed male C57BL/6J mice, BBR reduced inguinal and perirenal WAT mass while increasing O_2_ consumption, CO_2_ production, and energy expenditure. Using micro 18F-FDG PET/CT imaging, BBR increased BAT standardized uptake value, BAT volume, and total 18F-FDG uptake, with no clear increase in thermogenic activity in iWAT or eWAT. BBR also increased brown fat-enriched genes, including Ucp1, Prdm16, Elovl3, Ppargc1a, and Ppargc1b, while lowering plasma insulin and improving glucose metabolism. These findings suggest that BBR increases energy expenditure primarily by enhancing BAT recruitment and activation in HFD-fed mice [71].

Stearoyl-CoA desaturase 1 (SCD1), a key enzyme in lipid metabolism, was upregulated in HFD-fed mice but reduced following BBR administration, which contributed to decreased hepatic triglyceride accumulation by BBR. Similarly, the HFD-induced upregulation of upstream lipogenic regulators, including SREBP-1 and FAS, was reduced with BBR. The BBR-treated mice had improved insulin sensitivity and glucose tolerance. Collectively, these findings indicate the potential for BBR to attenuate hepatic steatosis [45].

Mi et al. [72] examined whether BBR attenuates insulin resistance in HFD/STZ-induced T2DM rats. BBR reduced FBG, FINS, IRI, total cholesterol, LDL-C, urine and serum corticosterone, plasma ACTH, pituitary ACTH, and hypothalamic Orexin-A, OX2R, and CRH protein levels. BBR also increased ISI, HDL-C, and skeletal muscle *GLUT4* mRNA and protein expression. These results demonstrate that BBR improves glucose and lipid metabolism in T2DM rats partly by suppressing HPA-axis hyperactivity and restoring skeletal muscle GLUT4 expression [72].

In male C57BL/6J mice, BBR prevented HFD-induced body weight gain and reduced WAT weight, eWAT volume, body fat percentage, serum lipids, glucose, and insulin levels. In eWAT, BBR downregulated Gal-3 expression, increased let-7d levels, and suppressed adipocyte differentiation and proliferation. These findings suggest that BBR reduces adiposity partly through let-7d-mediated downregulation of Gal-3, limiting adipocyte expansion in HFD-induced obesity [32].

InHFD-fed male C57BL/6J mice, BBR treatment significantly alleviated HFD-induced increased body and white adipose tissue weight, plasma triglyceride and blood glucose levels and expression of IL-6 and MCP-1. BBR also enhanced anti-inflammatory signaling in adipose tissue, as shown by increased M2 macrophage markers, including IL-4, CD206, and ARG1, and reduced macrophage infiltration partly associated with decreased CD68. These findings demonstrate the potential of BBR in acting as both an anti-obesity and anti-inflammatory agent [73].

In HFD-induced obese mice, BBR improved glucose tolerance and insulin sensitivity while reducing fasting glucose, fasting insulin, HOMA-IR, body weight, eWAT weight, liver weight, hepatic triglyceride content, ALT, AST, and hepatic lipid accumulation. In eWAT, BBR increased SIRT1 expression and activated LKB1–AMPK–ACC and AKT signaling, while reducing MCP-1, IL-6, TNF-α, p-JNK, p-IKKβ, and F4/80+ macrophage accumulation. BBR also decreased PGC-1α acetylation, and these effects were weakened in Sirt1+/− mice, suggesting that BBR attenuates insulin resistance and improves adipose inflammation and hepatic steatosis partly through SIRT1-dependent AMPK activation and suppression of inflammatory signaling [35].

In another study, inHFD-fed male C57BL/6J mice, BBR reduced body weight, blood glucose, total cholesterol, triglycerides, LDL-C, and the iWAT/body weight ratio while increasing the BAT/body weight ratio, energy expenditure, VO_2_, and VCO_2_. In adipose tissue, BBR reduced lipid droplet size in BAT and iWAT and increased UCP1 expression. These findings suggest that BBR improves HFD-induced obesity by activating AMPK/SIRT1 signaling, increasing PPARγ deacetylation, and promoting thermogenic adipose remodeling [36].

Li et al. [38] found that in HFD-fed male C57BL/6J mice, BBR reduced body weight, adipose tissue mass, liver weight, fasting blood glucose, serum triglycerides, total cholesterol, LDL-C, NEFA, AST, ALT, hepatic fibrosis, and hepatic lipid accumulation while increasing HDL-C and insulin sensitivity. In eWAT, BBR decreased adipocyte size, inflammatory markers including iNOS, COX-2, TNF-α, IL-1β, and IFN-γ, and chemokines including MCP-1, MIP-1α, Ccl5, Ccl11, Cx3cl1, and Cxcl10. BBR also increased M2 macrophage markers CD206 and CD301, reduced TNF-α-induced chemotaxis, and decreased macrophage-derived miR-155-5p. These results indicate that BBR attenuates insulin resistance and improves adipose inflammation, dyslipidemia, and liver injury through SIRT3-mediated inhibition of MAPK/NF-κB signaling and reduced inflammatory adipose–liver crosstalk [38].

Xia et al. [74] reported that BBR administration attenuated insulin resistance in HFD-fed male C57BL/6N mice. Body weight and hepatic lipid accumulation were both ameliorated with BBR over four weeks. BBR improved lipid metabolism by reducing serum free fatty acids (FFAs), triglycerides, and cholesterol. Intraperitoneal glucose tolerance (IPGTT) and intraperitoneal insulin tolerance tests (IPITT) suggested improved glucose homeostasis with BBR treatment. Aggravated hepatic hypoxia and elevated HIF-2α expression induced by HFD were reduced by BBR. BBR also restored hepatic glycogen stores, which were depleted with HFD and increased phosphorylation of AKT, GSK3*β*, and PP2A, indicating enhanced hepatic glycogen synthesis [74].

Ye et al. [53] used HFD-fed male C57BL/6 mice to examine whether BBR attenuates MAFLD-associated metabolic dysfunction through circadian clock regulation. Mice were fed a 60% HFD for 12 weeks, with BBR administered at 200 mg/kg/day during the final 4 weeks. BBR reduced fasting blood glucose, serum insulin, triglycerides, LDL-C, ALT, AST, hepatic lipid accumulation, and NAS score, while improving glucose tolerance and insulin sensitivity. BBR also reduced hepatic ROS and H_2_O_2_ levels and restored Bmal1 and Clock expression. These findings suggest that BBR attenuates HFD-induced hepatic metabolic dysfunction, oxidative stress, and insulin resistance partly through restoration of Bmal1/Clock-mediated circadian regulation [53].

Wang et al. [75] found that in HFD-fed male C57BL/6J mice, BBR improved glucose tolerance and insulin sensitivity, lowered fasting blood glucose, and reduced serum triglycerides, total cholesterol, LDL-C, hepatic triglycerides, and hepatic cholesterol. BBR also decreased liver weight, hepatic lipid accumulation, ALT, and AST levels, while increasing SIRT1 expression and reducing CPT1A expression. These results demonstrate that BBR improves hepatic steatosis, liver injury, and systemic lipid metabolism in HFD-induced metabolic dysfunction [75].

Cheng et al. [55] used a high-fructose-fed rat model and found that BBR reduced body weight gain, fasting blood glucose, serum ALT and AST, hepatocyte necrosis, vacuolation, and inflammatory infiltration, while improving glucose tolerance and insulin sensitivity. BBR also reduced serum and hepatic IL-6, TNF-α, ROS, and MDA levels, restored IL-10, SOD, and GSH levels, and increased hepatic ADK, p-AMPK, Nrf2, and HO-1 expression. These findings suggest that BBR attenuates fructose-induced hepatic injury, inflammation, oxidative stress, and insulin resistance partly through activation of the ADK/AMPK/Nrf2 pathway [55].

Yang et al. [56] used HFD-fed C57BL/6J mice and administration of BBR reduced body weight, serum and hepatic triglyceride levels, fasting glucose, random glucose, and hepatic steatosis without significantly altering food intake. BBR also reduced hepatic AKR1B10 expression, while increasing PPARα, CPT-1, GK, and the p-GSK3β/GSK3β ratio and decreasing PPARγ, GLUT2, PEPCK, and G6Pase expression. In AKR1B10 knockdown mice, the metabolic and PPAR-related effects of BBR were weakened, suggesting that BBR improves HFD-induced hepatic lipid accumulation and glucose metabolic dysfunction partly through AKR1B10-dependent regulation of PPAR signaling [56].

In HFD-fed C57BL/6 mice, BBR reduced body weight, adipocyte size, skeletal muscle lipid droplet accumulation, TNFα and IL-6 expression, and the atrophy markers FBXO32 and MuRF-1. BBR also improved glucose tolerance, increased *LC3B* mRNA expression, and restored FUNDC1 protein levels. These findings demonstrate that BBR protects against obesity-induced skeletal muscle atrophy partly by reducing inflammation and lipid accumulation while promoting FUNDC1-related mitophagy [27].

BBR reduced weight gain, ALT, AST, hepatic inflammation, lipid accumulation, hepatic triglycerides, hepatic total cholesterol, and WAT size, while improving glucose tolerance and insulin sensitivity in HFD-fed male C57BL/6J mice [58] BBR also reduced lipogenic genes including *Fas*, *Scd1*, and *Nr1h3*, while increasing *Cpt1α* expression, suggesting reduced hepatic lipogenesis and enhanced fatty acid oxidation. BBR increased p-PI3K and p-Akt while decreasing p-TBK1/TBK1 and p-IRF3/IRF3 ratios, indicating suppression of STING downstream signaling. These findings suggest that BBR attenuates HFD-induced MASLD partly by activating PI3K/Akt signaling and inhibiting STING-mediated inflammatory and metabolic dysfunction [58].

The HFD- induced hepatic triglyceride content and lipid droplet formation in C57BL/6J mice, were attenuated by BBR indicating its anti-steatotic effects. Hepatic lipid-metabolism proteins, including CIDEA and PLIN4, were overexpressed in HFD-fed mice, an effect that was significantly suppressed by BBR treatment [76].

Wong et al. [77] investigated the effects of Berberine ursodeoxycholate (HTD1801), in male Syrian golden hamsters fed a high-fat, high-cholesterol diet and found significantly reduced serum ALT, AST, LDL-C, and total cholesterol levels with BBR administration. Histological analyses further demonstrated reductions in hepatic steatosis, inflammation, and fibrosis, indicating attenuation of MASH progression. Hepatic transcriptomic analyses suggested that BBR regulated pathways involved in lipid metabolism, inflammation, and extracellular matrix remodeling, supporting its hepatoprotective effects [77].

Li et al. [59] used HFD-fed male C57BL/6J mice to examine whether combined metformin and BBR attenuates NAFLD. BBR and metformin alone partially improved obesity-associated metabolic dysfunction, while the combination produced stronger reductions in hepatic-to-body mass ratio, visceral fat mass, fasting glucose, insulin, HOMA-IR, liver size, hepatic lipid accumulation, hepatic triglycerides, hepatic cholesterol, ALT, AST, TNF-α, IL-1β, and IL-6. Combined treatment also improved serum lipid profiles by increasing HDL-C and reducing LDL-C, triglycerides, and total cholesterol, and significantly lowered NAS score. The combined treatment increased hepatic p-AMPK/AMPK and suppressed SREBP1 and FASN, suggesting that the combination attenuates HFD-induced NAFLD through AMPK activation and downstream inhibition of lipogenesis [59].

Alpaslan et al. [78] evaluated whether free BBR and liposomal/nano-BBR promote adipose tissue browning in female C57BL/6J mice fed a 60% HFD. Both BBR formulations increased PRDM16 and CIDEA expression in WAT and BAT. These findings demonstrate that free and nano-encapsulated BBR may partially induce adipose browning markers, although the protein-level evidence for functional thermogenesis was limited [78].

### 3.3. Effects of Berberine on Other Animal Models of Diabetes

Zhang et al. [79] assessed whether BBR could limit hepatic steatosis progression in male db/db mice Table 6. BBR attenuated insulin resistance and reduced body weight, hepatic lipid accumulation, hepatic triglycerides, total cholesterol, LDL, ALT, and AST after five weeks of treatment. BBR also inhibited hepatic lipoperoxide accumulation, downregulated lipogenic genes including *SREBP-1c*, *ChREBP*, *FAS*, and *C/EBPβ*, and reduced *CYP2E1* and *CYP4A10* mRNA expression. Increased catalase expression further suggests that BBR attenuates diabetic hepatic steatosis partly by suppressing lipogenesis, lipid peroxidation, and oxidative stress [79].

BBR treatment of male C57BL/KsJ-db/db mice reduced fasting blood glucose, HbA1c, and glucagon levels, while restoring serum insulin and increasing hepatic glycogen content. Metabolomic analyses showed reductions in most glucose-related metabolites, but hepatic G-6-P/G-1-P increased, supporting enhanced hepatic glucose phosphorylation. BBR also increased hepatic GK expression*, HK4/GCK* and *HK2* mRNA levels, and GLUT2 protein expression, while reducing PCK1 expression. BBR did not substantially alter GKRP expression, but reduced the GK-GKRP binding ratio, suggesting that BBR increases functional hepatic GK availability by promoting GK release from GKRP [44].

Zhong et al. [80] used ob/ob mice and STZ-induced diabetic C57BL/6J mice and found reduced fed blood glucose and fasting plasma insulin in ob/ob mice, decreased fasting blood glucose in STZ-induced diabetic mice, and improved glucose tolerance and insulin sensitivity with BBR administration. BBR also suppressed lactate-induced hepatic glucose production and reduced hepatic *Pepck*, *G6pase*, and *PGC1α* mRNA expression in both diabetic models. BBR reduced glucagon-stimulated blood glucose, hepatic cAMP levels, and p-CREB expression, suggesting inhibition of the glucagon–cAMP–CREB pathway. These results demonstrate that BBR attenuates diabetic hyperglycemia partly by suppressing glucagon-driven hepatic gluconeogenesis rather than acting solely through insulin-dependent mechanisms [80].

Meng et al. [24] used HFD/STZ diabetic rats and DEX-treated mice. In diabetic rats, BBR and HGSD, a BBR analogue, reduced fasting blood glucose and IPGTT AUC, increased insulin-stimulated GLUT4 translocation, and restored IRS1 and AKT phosphorylation in skeletal muscle. HGSD also reduced skeletal muscle corticosterone levels and produced stronger effects than equivalent-dose BBR. In DEX-treated mice, BBR and HGSD lowered fasting glucose, fasting insulin, and OGTT AUC while increasing insulin-stimulated GLUT4 translocation, p-IRS1, and p-AKT. Co-immunoprecipitation analyses showed that HGSD increased PI3K association with p-IRS1 and reduced PI3K association with GR/GRα, suggesting that HGSD improves skeletal muscle insulin signaling by limiting glucocorticoid receptor interference with the IRS1–PI3K–Akt–GLUT4 pathway [24].

Yao et al. [25] examined whether BBR could reduce ectopic skeletal muscle lipid deposition in male db/db mice. BBR reduced weight gain, fasting blood glucose, insulin, serum FFA, serum and skeletal muscle triglycerides, and cholesterol levels. In gastrocnemius muscle, BBR increased mitochondrial number, citrate synthase activity, mitochondrial biogenesis regulators PGC-1α, ERRα, NRF-1, and mtTFA, mitochondrial function genes *COXII*, *COXIV*, and *CytC*, and the lipid metabolism gene *CPT1β*. Increased AMPK phosphorylation suggests that BBR protects against skeletal muscle lipid accumulation and mitochondrial dysfunction through AMPK–PGC-1α-mediated mitochondrial biogenesis [25].

Li et al. [81] investigated the anti-hyperglycemic effects of BBR in male C57BL/6 J wild-type mice and leptin receptor-mutated (C57BL/KsJ-db/db) mice. The db/db mice were spontaneously hyperglycemic; however, four weeks of BBR treatment significantly reduced blood glucose levels. Lipid profiles were analyzed in db/db and BBR-treated db/db mice. BBR significantly lowered serum LDL-C and triglyceride levels compared with untreated db/db mice [81].

Dong et al. [82] used an acute intralipid infusion model with euglycemic-hyperinsulinemic clamps to determine whether BBR could attenuate lipid-induced insulin resistance in male Sprague–Dawley rats. BBR increased steady-state glucose infusion rate without altering blood glucose, indicating improved insulin sensitivity independent of changes in glycemia. In gastrocnemius muscle, BBR reduced intralipid-induced mitochondrial swelling and suppressed CypD protein expression while increasing p-AKT and p-AMPK levels. These findings suggest that BBR attenuates acute lipid-induced insulin resistance primarily through skeletal muscle effects involving reduced CypD-mediated mitochondrial dysfunction and activation of AMPK/AKT signaling [82].

Yang et al. [83] used tunicamycin-treated C57BL/6 mice to examine whether BBR protects against ER stress-induced hepatic injury and metabolic disruption. BBR improved liver gross morphology and histology, reduced hepatic triglyceride accumulation, increased *CD36* mRNA expression, and decreased tunicamycin-induced serum and hepatic FGF21 levels. BBR also increased insulin-stimulated p-AKT while reducing TRB3, CHOP, GRP78, ATF6, PERK, p-eIF2α, and IRE1α signaling, suggesting reduced ER stress and improved hepatic insulin signaling. These results indicate that BBR attenuates acute ER stress-induced hepatic metabolic dysfunction partly by suppressing UPR activation and modulating gut microbiota, although the study is more of a supporting liver-injury mechanism than a core insulin-resistance model [83].

Xu et al. [50] found that in db/db mice, BBR improved glucose tolerance and insulin sensitivity while reducing fasting blood glucose, triglycerides, total cholesterol, LDL-C, and hepatic lipid accumulation. BBR also reduced hepatic PEPCK and G6Pase expression, increased SIRT1 and Opa1 protein expression, decreased Drp1 expression, and improved mitochondrial morphology. These findings suggest that BBR attenuates hepatic insulin resistance in db/db mice partly by restoring SIRT1/Opa1-mediated mitochondrial dynamics and reducing hepatic lipid and glucose metabolic dysfunction [50].

Lu et al. [52] used spontaneous diabetic rats. BBR reduced hepatic glucose production and downregulated the gluconeogenic enzymes G6Pase and PEPCK. BBR increased p-AKT1, NO, cGMP, and PKG signaling while reducing p-CREB expression. These in vivo findings support the primary hepatocyte results, suggesting that BBR inhibits diabetic hepatic gluconeogenesis by activating AKT1/MAPK-dependent NO/cGMP/PKG signaling and suppressing CREB-mediated gluconeogenic enzyme expression [52].

Chen et al. [54] used HFD/STZ-induced T2D mice. BBR reduced body weight, fasting blood glucose, serum insulin, liver weight, eWAT weight, hepatic lipid accumulation, hepatic TG and FFA, serum TG and TC, ALT, AST, and ALP. BBR also improved glucose tolerance and insulin sensitivity, increased hepatic p-AKT, p-AMPKα, PPARγ, FGF21, and GLUT2 expression, and increased pancreatic GLUT2 expression. In addition, BBR increased VO_2_, VCO_2_, respiratory exchange ratio, and energy expenditure, improved pancreatic islet morphology, and reduced *IL-1β* mRNA levels in liver, eWAT, skeletal muscle, and pancreas. These effects were diminished in FGF21 knockout mice, suggesting that BBR attenuates T2D-associated insulin resistance and protects against hepatic lipid accumulation, inflammation, and liver/pancreatic injury partly through FGF21-dependent activation of PPARγ–FGF21–GLUT2 signaling [54].

Cheng et al. [84] used both DIO C57BL/6J mice and leptin-deficient ob/ob mice. BBR reduced body weight, fat mass, adipocyte size, hepatic lipid deposition, serum insulin, triglycerides, total cholesterol, and free fatty acids while improving glucose tolerance. In adipose tissue, BBR increased capillary density, CD31 and Ki67 expression, CD34+CD31+ endothelial progenitor cells, VEGFA/VEGFR2, mitochondrial abundance, and browning markers including PPAR-γ, PRDM16, and UCP1. Transcriptomic analyses identified GPx3 as a BBR-responsive target, suggesting that BBR restores adipose angiogenesis and beige adipogenesis through GPx3-mediated suppression of chronic HIF-1α activation and activation of PRDM16 signaling [84].

**Figure 4:**
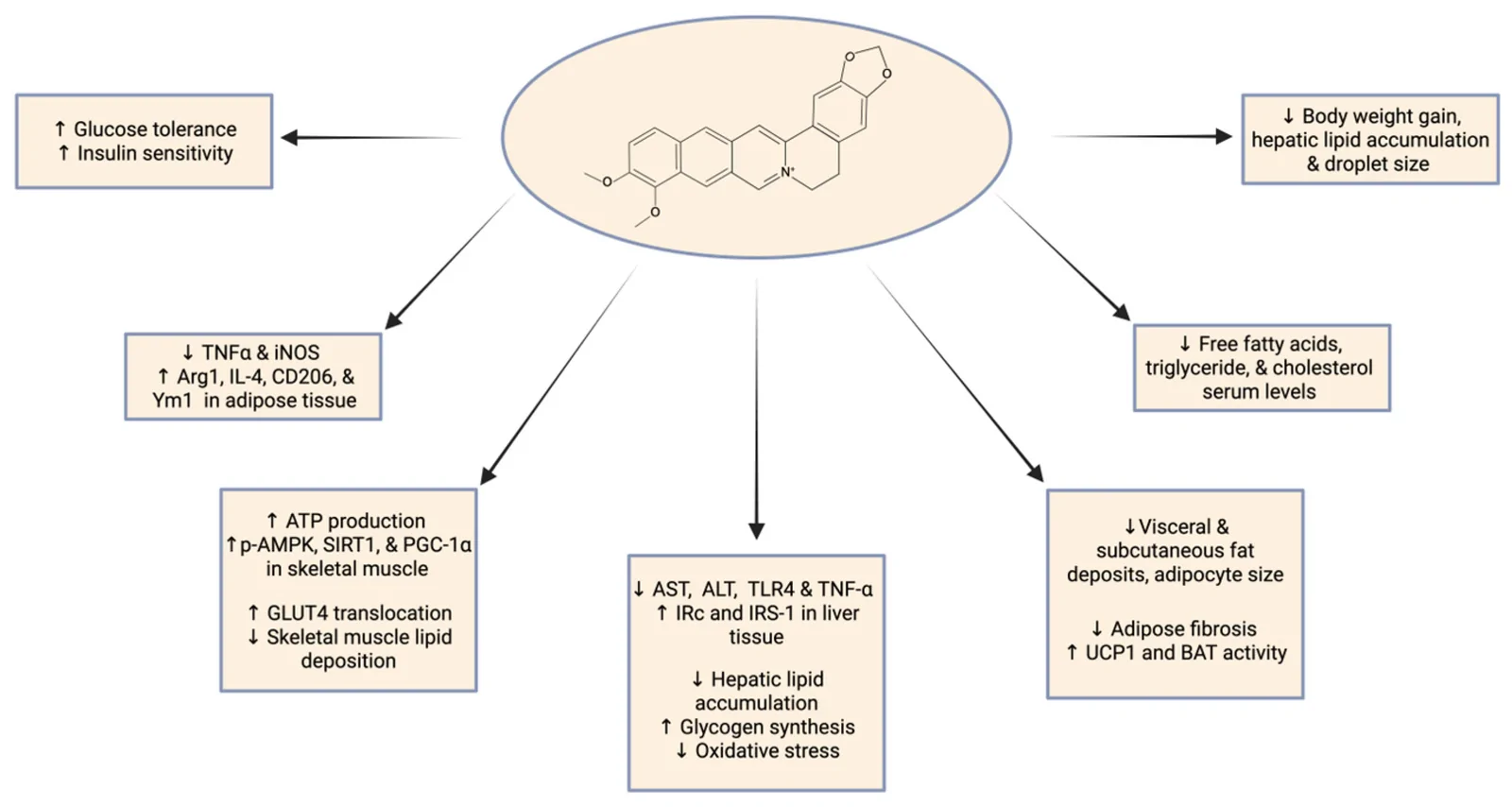
Summary of the Effect of Berberine in Insulin-Sensitive Tissues of Animals. Created in BioRender. Harrison, M. (2026) https://BioRender.com/qwbblzg.

Across the in vivo studies, BBR was administered at doses ranging from 1.5 to 300 mg/kg/day through oral or intraperitoneal delivery, with one additional study using intravenous free or nano-encapsulated BBR. Its effects were evaluated in HFD-induced, age-related, and genetically induced insulin-resistant models. Overall, BBR reduced diet- and genotype-induced weight gain and lowered serum free fatty acids, triglycerides, and cholesterol. BBR also decreased hepatic lipid accumulation and lipid droplet size, accompanied by a reduction in liver injury markers ALT and AST. Systemic insulin sensitivity and glucose tolerance improved across models, and skeletal muscle ATP production increased in association with upregulation of p-AMPK, SIRT1, and PGC-1α. In adipose tissue, BBR reduced pro-inflammatory markers and increased anti-inflammatory signaling. Visceral and subcutaneous fat mass, along with adipocyte size, were significantly reduced with BBR treatment (Figure 4).

## 4. Clinical Effects of Berberine in Humans with Metabolic Disease

Although most evidence for BBR’s effects on insulin-sensitive tissues comes from preclinical models, several human clinical trials have evaluated its metabolic effects in obesity, NAFLD/MASLD, T2D, and related metabolic disorders.

Yan et al. [62] investigated the effects of BBR in adults with NAFLD Table 7. Participants were middle-aged males and females (BMI 24–30 kg/m^2^) and received 1500 mg of BBR daily for 16 weeks. BBR combined with lifestyle intervention (LSI, dietary modifications and exercise) significantly decreased hepatic fat content compared with LSI alone. Serum ALT and AST levels were also decreased in the BBR + LSI group. Compared with LSI alone, BBR significantly reduced body weight, BMI, and waist circumference after 16 weeks, indicating its ability to improve energy metabolism. Blood glucose levels were also decreased substantially within four weeks of BBR + LSI treatment compared to baseline [62].

Chang et al. [85] extended the Yan et al. [62] clinical trial by performing serum lipidomic profiling in patients with NAFLD and found reductions in fatty acids, ceramides, sphingomyelins, lysophosphatidylinositols, phosphatidylinositols, and lysophosphatidylcholines with BBR administration. These findings suggest that BBR attenuates NAFLD partly by altering lipid species linked to hepatic steatosis and insulin resistance [85].

Di Bisceglie et al. [86] conducted a phase 1b dose-ranging study evaluating the pharmacokinetics, pharmacodynamics, and safety of an analogue of BBR (HTD1801), in adults with hyperlipidemia. BBR significantly reduced triglycerides, total cholesterol, LDL-C, and non-HDL cholesterol in a dose-dependent manner and was generally well tolerated [86].

Rondanelli et al. [87] administered berberine phytosome (1100 mg/day) to females with polycystic ovary syndrome (PCOS) and found that BBR reduced HOMA-IR, fasting glycemia, insulin, VLDL, triglycerides, testosterone, CRP, and TNF-α, while increasing SHBG. Anthropometric measurements showed significant reductions in BMI, waist circumference, hip circumference, waist-to-hip ratio, visceral adipose tissue, total body mass, and fat mass. Improvements in acne severity were also observed, indicating beneficial effects on metabolic, inflammatory, and hyperandrogenic features associated with PCOS [87].

Harrison et al. [88] evaluated the effects of berberine ursodeoxycholate (HTD1801) in males and females aged 26–75 years with presumed non-alcoholic steatohepatitis (NASH) and type 2 diabetes. Participants with a BMI of 25–55 kg/m^2^ received oral HTD1801 at doses of 500 mg or 1000 mg twice daily for 18 weeks. HTD1801 significantly reduced liver fat content, HbA1c, ALT, GGT, LDL-C, triglycerides, and body weight compared with placebo, demonstrating suppression of hepatic steatosis and improvements in glycemic control, lipid metabolism, and liver function [88].

Rondanelli et al. [89] administered BBR Phytosome™ (550 mg/twice daily for 60 days) to overweight males and females and found that BBR reduced glycemia, total cholesterol, insulin, ApoB, and ALT levels compared to baseline. Anthropometric measurements showed decreases in waist circumference, visceral adipose tissue, and fat mass in the BBR group. Between-group comparisons showed significant reductions in glycemia, total cholesterol, triglycerides, insulin, visceral adipose tissue, and fat mass in the BBR group compared with placebo [89].

Panigrahi et al. [90] evaluated the effects of HIMABERB^®^, a BBR formulation, in males and females aged 18–55 years with prediabetes. BBR significantly reduced fasting plasma glucose, fasting insulin, 2 h oral glucose tolerance test (2 h OGTT) glucose, HOMA-IR, and HbA1c, demonstrating improvements in glycemic control and insulin sensitivity. Treatment also significantly decreased the liver enzymes ALP, AST, and ALT, indicating improved hepatic function [90].

Koperska et al. [91] assessed BBR supplementation in middle-aged males and females (BMI 25.8–39.9 kg/m^2^) with MAFLD. Trunk fat mass was significantly reduced in the BBR group compared with placebo. ALT levels were significantly reduced in the BBR group, whereas AST levels did not differ between groups. Liver function assessed using the De Ritis ratio, which is the ratio of AST/ALT, showed a decrease from baseline in the BBR group. Total cholesterol levels were significantly lower in the BBR group than in the placebo group. These findings indicate that BBR may improve metabolic function in patients with MAFLD [91].

Bandala et al. [92] administered BBR to obese male and female participants. The effects of BBR were compared with *Gymnema sylvestre* (GS). Over the treatment period, BBR significantly decreased Visfatin and Apelin (Ap) levels and increased levels of Omentin (Ome). Anthropometric measurements showed that BBR significantly decreased body weight, BMI, body fat percentage, visceral fat percentage, and diastolic blood pressure compared with baseline. Compared with GS, BBR significantly decreased BMI, waist-hip ratio (WHR), and both systolic and diastolic blood pressure. The findings demonstrate the beneficial effect of BBR on body composition in obese individuals [92].

Wong et al. [77] reported a secondary analysis of the phase II randomized clinical trial previously published by Harrison et al., evaluating HTD1801 in patients with metabolic dysfunction-associated steatohepatitis (MASH). Consistent with the original publication, HTD1801 reduced liver fat content, body weight, and ALT levels. The additional histologic analyses further demonstrated increased rates of MASH resolution, providing further evidence for the hepatoprotective effects of HTD1801 [77].

Ji et al. [93] investigated the effects of HTD1801 in males and females with type 2 diabetes. HTD1801 significantly reduced HbA1c, fasting plasma glucose, HOMA-IR, and fasting insulin levels, indicating improved glycemic control and insulin sensitivity. HTD1801 also reduced total cholesterol, LDL-C, ALT, AST, and GGT, suggesting additional improvements in lipid metabolism and liver-related markers [93].

Tu et al. [94] evaluated BBR and dihydroberberine (dhBBR) in patients with cholestatic liver disease. Participants received 500 mg BBR or dhBBR three times daily for 15 days, which reduced GGT, ALP, ALT, AST, and circulating 5-hydroxytryptamine (5-HT) levels. Mechanistic analyses suggested that gut microbiota metabolize BBR into dhBBR, which suppresses intestinal enterochromaffin cell 5-HT production and improves bile acid homeostasis. These findings identify the gut microbiota–dhBBR–5-HT axis as a potential mechanism by which BBR attenuates cholestatic liver injury [94].

**Figure 5:**
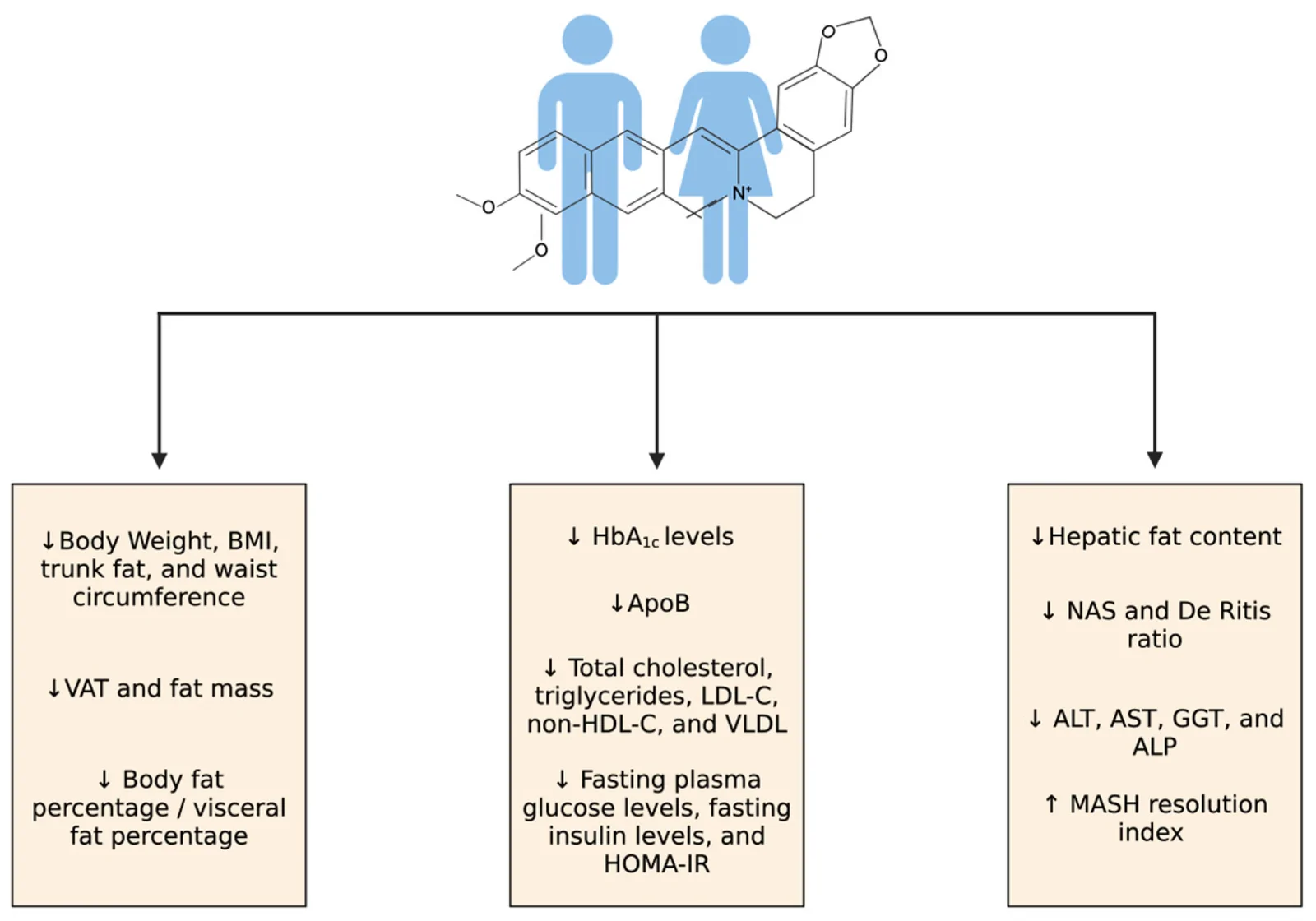
Summary of the Effect of Berberine in Human Clinical Studies. Created in BioRender. Harrison, M. (2026) https://BioRender.com/4e1pamc.

Lei et al. [95] conducted a multicenter, randomized, double-blind, placebo-controlled trial evaluating berberine hydrochloride in adults with obesity and MASLD. BBR reduced LDL-C, ApoB, and hs-CRP, but did not significantly improve liver fat content, visceral adipose tissue area, or insulin resistance compared with placebo. In post hoc analyses, participants with higher baseline hs-CRP showed greater reductions in total cholesterol, LDL-C, ApoB, hs-CRP, BMI, waist-to-height ratio, waist circumference, and body weight. These findings suggest that BBR may reduce lipid and inflammatory markers, with broader metabolic effects potentially influenced by baseline inflammatory status [95] (Figure 5).

## 5. Summary/Conclusions

In summary, in vitro studies show that BBR increases glucose uptake in myocytes by increasing AMPK phosphorylation. In adipocytes, BBR reduces preadipocyte differentiation and lipid accumulation by decreasing key pro-adipogenic genes, including PPARγ, C/EBPα, and their downstream effectors. Similar to its effects in myocytes, BBR increases glucose uptake through improved glucose transport in adipocytes. Furthermore, BBR modulates insulin-related IRS-1/PI3K/AKT signaling while reducing inflammatory kinase activation, including IKKβ/NF-κB-related signaling. In hepatocytes, BBR decreases gluconeogenesis by suppressing regulators of hepatic glucose production, including HNF4α and downstream enzymes such as PEPCK and G6Pase. Additionally, BBR reduces lipid accumulation by activating AMPK and downregulating pro-lipogenic proteins such as SCD1, ChREBP, and FAS.

In vivo study findings demonstrated that BBR reduces HFD-induced body-weight gain, attenuates insulin resistance, and increases glucose tolerance through coordinated actions in skeletal muscle, adipose tissue, and the liver. In the liver, BBR reduces lipid accumulation and lipid droplet size by lowering hepatic triglyceride content and downregulating lipid-droplet-associated markers including Cidea and Plin4. BBR also decreases liver injury markers, ALT and AST, indicating hepatoprotective effects. In diabetic animals, BBR increases mitochondrial number and reduces triglyceride accumulation in muscle tissue. BBR attenuates age-related insulin resistance by decreasing fasting glucose, insulin, and serum triglyceride levels. Specifically, BBR enhances GLUT4 translocation, AMPK phosphorylation, activation of SIRT1, PGC-1α, and ATP production in muscle tissue. BBR also improves lipid profiles by reducing serum triglycerides, FFAs, and cholesterol levels. Additionally, BBR reduces both subcutaneous and visceral fat deposits, consistent with histological evidence of smaller adipocyte size. These reductions are partly attributable to BBR-induced upregulation of UCP1, which increases BAT activity and energy expenditure. BBR also reduces macrophage infiltration into adipose tissue and shifts inflammatory signaling by upregulating anti-inflammatory and downregulating pro-inflammatory markers.

Clinical studies have evaluated BBR and BBR-based formulations in individuals with NAFLD/MASLD, obesity, prediabetes, T2DM, PCOS, and dyslipidemia. Across trials, BBR consistently improved lipid metabolism, with reductions in triglycerides, total cholesterol, LDL-C, non-HDL-C, ApoB, and VLDL. Several studies also reported reductions in body weight, BMI, waist circumference, visceral adiposity, trunk fat, and fat mass, suggesting benefits on adiposity and body composition. In populations with liver-focused metabolic dysfunction, BBR reduced hepatic fat content, ALT, AST, GGT, ALP, or De Ritis ratio, supporting potential hepatoprotective effects in NAFLD/MASLD. BBR also improved glycemic control and insulin sensitivity in several studies, including reductions in fasting glucose, fasting insulin, HOMA-IR, HbA1c, and OGTT glucose responses. Inflammatory markers were less consistently measured, but reductions in CRP, hs-CRP, and TNF-α suggest possible anti-inflammatory effects.

However, the clinical evidence is heterogeneous because several trials used BBR-derived formulations such as BBR phytosome, HIMABERB, dhBBR, or HTD1801/BBR ursodeoxycholate, and the largest BBR hydrochloride trial improved LDL-C, ApoB, and hs-CRP, with broader anthropometric effects observed mainly in post hoc analyses among participants with higher baseline hs-CRP. Together, these findings suggest that BBR has the strongest clinical support for improving dyslipidemia, adiposity, liver enzyme profiles, and glycemic markers, while its effects on hepatic fat and insulin resistance may depend on population, formulation, dose, and treatment duration.

Several in vitro studies support a functional role for AMPK because pharmacological inhibition with Compound C or AMPK knockdown/knockout attenuated the BBR-induced effects on glucose uptake, adipocyte lipolysis, thermogenic remodeling, and hepatocyte lipid accumulation [26,29,34,36,46]. However, these studies were performed in cell models, and the reviewed animal studies did not use tissue-specific AMPK knockout models to test whether AMPK is required for BBR’s in vivo metabolic effects.

Although many of the reviewed studies show robust activation of AMPK in insulin-sensitive tissues in response to BBR, whether this activation is direct or a consequence of energy depletion is unknown.

Some studies suggest that AMPK activation may occur secondary to energy stress or mitochondrial effects, as BBR increased AMP and the AMP/ATP ratio in fructose-treated muscle and hepatocyte models [26,55], reduced mitochondrial respiration or ATP production [43,48], To better determine the role of AMPK on BBR action future studies should use tissue-specific AMPK knockdown or knockout models, and multi-tissue metabolic profiling.

The effects of parent BBR should also be distinguished from those of its metabolites because intestinal metabolism may contribute to its biological activity. This review is focused primarily on BBR and BBR-based interventions; however, in vivo BBR undergoes intestinal, hepatic, and microbiota-mediated metabolism before reaching insulin-sensitive tissues. Therefore, effects attributed to BBR may reflect the combined actions of parent BBR and metabolites such as berberrubine, demethyleneberberine, thalifendine, and jatrorrhizine. This distinction is important because metabolite-specific studies suggest that some metabolic effects attributed to BBR may involve active metabolites. Yang et al. further showed that berberrubine improved hepatic steatosis and insulin resistance in HFD-fed mice and OA-treated HepG2 cells, with some glucose-homeostasis outcomes exceeding those of parent BBR [22]. Sun et al. found that BBR and berberrubine reduced serum glucose in HFD-induced obese mice and decreased hepatic G6Pase and PEPCK expression through intestinal FXR-related signaling [96]. Therefore, metabolite-mediated effects do not negate the significance of BBR; rather, orally administered BBR produces both low systemic exposure to parent BBR and active metabolites that may collectively contribute to its metabolic effects.

The absorption, tissue exposure, and pharmacokinetics of BBR should also be considered when designing human studies and evaluating its potential clinical use. Orally administered BBR has low systemic bioavailability because of poor intestinal absorption, P-gp-mediated efflux, and intestinal and hepatic first-pass metabolism. Feng et al. [21] reported very low oral bioavailability of parent BBR in rats and identified multiple BBR metabolites, showing that systemic exposure after oral dosing may reflect both parent BBR and metabolite profiles. This complicates interpretation of cell studies because in vitro models directly expose skeletal muscle, adipocyte, or hepatocyte cells to parent BBR, whereas animal studies involve absorption, metabolism, tissue distribution, and microbiota-mediated conversion before BBR reaches insulin-sensitive tissues. Importantly, Tan et al. found that after oral administration of BBR at 200 mg/kg in rats, BBR distributed to metabolically relevant tissues, with overall exposure approximately 10-fold higher in liver, 5-fold higher in kidney, and 1.2-fold higher in muscle than in plasma [97]. Therefore, low circulating parent-BBR levels do not necessarily exclude tissue exposure, but animal findings should be interpreted in the context of dose, route, formulation, tissue accumulation, and metabolite formation. Future studies should measure parent BBR and metabolite levels in plasma and insulin-sensitive tissues alongside metabolic and insulin-signaling outcomes.

In this review, skeletal muscle cell studies used approximately 1–50 µM BBR, adipocyte studies used approximately 1–100 µM BBR, and hepatocyte studies used approximately 0.1–100 µM BBR. These concentrations are useful for identifying cellular pathways but may not reflect circulating parent BBR levels or tissue exposure achieved after oral dosing in humans. In a human pharmacokinetic crossover study, Yagiz et al. gave 12 healthy adults a single 800 mg oral BBR dose as either powder or emulsified with vitamin E TPGS or *Quillaja* extract to improve intestinal absorption [98]. Although TPGS and Quillaja increased parent-BBR plasma Cmax from 0.9 nM to 2.0 and 5.1 nM, respectively, these circulating levels remained far below the micromolar concentrations commonly used in cell models [98]. However, this concentration gap does not negate the value of in vitro evidence. Similar issues exist for established metabolic drugs such as metformin, where many in vitro studies use concentrations above 1 mM despite therapeutic human plasma concentrations of approximately 10–40 µM [15]. Therefore, in vitro BBR studies should be interpreted as mechanistic screening evidence that identifies BBR-responsive pathways and supports future work on translational effects.

Safety concerns and potential adverse effects of long-term BBR supplementation should be interpreted in the context of the relatively short durations studied clinically. In the included human studies, plain BBR was commonly administered as 500 mg twice daily for 6 months [95] or 500 mg three times daily for 12–16 weeks [62,91,92], with generally good short-term tolerability. Yan et al. [62] reported that BBR-related adverse events in 184 adults with NAFLD were mild and mainly digestive-system related, while Bandala et al. [92] reported gastrointestinal symptoms that attenuated during the first month of treatment. Panigrahi et al. [90] reported no severe adverse effects or liver/kidney toxicity after 12 weeks of HIMABERB in adults with prediabetes, and Lei et al. [95] found a similar incidence of adverse events between BBR and placebo after 6 months in 337 adults with obesity and MASLD. Although outside the primary 2015–2026 search window, one 2012 pilot study in obese adults receiving oral BBR at 500 mg three times daily for 12 weeks suggested no obvious short-term hepatic or renal toxicity signal under these conditions [99]. However, these studies lasted weeks to months rather than years. Therefore, long-term safety with chronic nutraceutical use remains unclear, and future trials should include longer follow-up, standardized adverse-event reporting, and liver and kidney function testing.

The relationship between gut microbiota and BBR action deserves further attention because microbiota may contribute to BBR efficacy through both metabolism and gut–liver signaling. Since orally administered BBR has low systemic bioavailability, intestinal bacteria may influence its activity by converting parent BBR into more absorbable or biologically active metabolites, including dhBBR. Several studies in this review support this interpretation. Sun et al. linked BBR-mediated improvements in hepatic lipid accumulation to altered microbial bile acid metabolism and intestinal FXR signaling [64]. Liu et al. showed that BBR reduced LPS–TLR4 inflammatory signaling in HFD-induced metabolic dysfunction [66], while Yang et al. linked BBR’s liver-protective effects during hepatic ER-stress injury to gut microbiota modulation [83]. Tu et al. further showed that gut microbiota converted BBR to dhBBR, which suppressed intestinal 5-HT production and improved cholestatic liver injury [94]. Together, these findings suggest that some systemic effects of oral BBR may originate from intestinal metabolism, microbial signaling, and gut–liver communication rather than direct exposure of insulin-sensitive tissues to parent BBR alone. Future studies should integrate microbiome profiling and tissue-specific metabolic outcomes to clarify how intestinal mechanisms contribute to BBR’s effect in skeletal muscle, adipose tissue, and liver.

Potential effects of BBR on appetite and central regulation of food intake also remain unclear. Guo et al. [63] assessed HFD mice receiving oral BBR at 100mg/kg/day for 4 weeks, and Yang et al. [56] assessed HFD mice receiving oral BBR at 50mg/kg/day for 4 weeks, and both reported metabolic improvements without changes in food intake. Direct assessment of appetite, taste-related signaling, or central appetite pathways remains limited. Future studies should determine whether these mechanisms contribute to BBR-mediated changes in body weight and metabolism.

Potential harmful effects of the animal dosing regimens should be interpreted by delivery route. Although the full dose range appears large, most orally administered BBR studies used 5–300 mg/kg/day, while intraperitoneal studies used lower doses of 1.5–10 mg/kg/day [71,73,84]. Oral studies at the upper end of this range, including 300 mg/kg/day for 4 weeks in HFD-fed mice [45] and 75–300 mg/kg/day for 3 days in tunicamycin-induced liver injury [83], reported improved hepatic steatosis, glucose tolerance, insulin resistance, liver injury markers, or ER-stress markers rather than overt harmful effects. Intraperitoneal studies using 1.5 mg/kg/day for 4–6 weeks or 5–10 mg/kg/day for 4 weeks also reported improved adipose, lipid, glucose, and inflammatory outcomes [71,73,84]. However, most studies were designed to test metabolic efficacy rather than systemic toxicity, and the absence of reported harm does not establish long-term safety. Future animal studies should include dose–response toxicity assessment, liver and kidney function markers, and histological safety endpoints.

Current preclinical studies remain limited by variable animal models, BBR doses and administration routes, and inconsistent assessment of insulin sensitivity. Future studies should use standardized insulin-sensitivity measurements and better define dose- and route-dependent effects. More systematic safety assessment is also needed, including liver and kidney function, tissue histology, and evaluation of overall toxicity. Finally, pharmacokinetic studies examining circulating BBR, tissue exposure, and metabolite levels are needed to better relate administered dose to biological effects.

Current human studies remain limited by variable BBR doses, treatment durations, and formulations, as well as inconsistent assessment of insulin sensitivity. For example, plain BBR was commonly administered at 500 mg twice or three times daily, while studies also used BBR phytosome (550 mg twice daily), HIMABERB (500 mg three times daily), dhBBR, and HTD1801 (500–1000 mg twice daily). Future trials should compare multiple doses of the same formulation within the same study and directly compare plain BBR with higher-bioavailability formulations using standardized treatment durations and insulin-sensitivity measurements. Longer-term safety studies should include adverse-event monitoring and liver and kidney function assessment. Finally, pharmacokinetic studies measuring circulating BBR and its metabolites are needed to relate dose and formulation to systemic exposure and metabolic effects. Combination studies are also warranted, particularly with established therapies such as metformin, as well as other well-studied phytochemicals, to determine whether BBR can complement existing metabolic interventions. Clarifying these factors will be essential for optimizing BBR as a targeted metabolic modulator of insulin-sensitive tissues.

## Figures and Tables

**Table 1:** Effects of Berberine on Skeletal Muscle cells (in vitro).

Source	Cell Type	Treatment	Findings
[23]	C2C12 Myoblasts	BBR 10 μM for 24 h IL-1β 10 ng/mL and/or TNF-α 50 ng/mL	↓ Nuclear translocation of NF-κB ↓ p-SAPK/JNK protein levels ↓ C-JUN and SMAD4 expression ↓ Nuclear translocation of p38 MAPK ↑ Catalase protein levels ↓ iNOS expression ↑ AMPK, PGC-1α, SIRT1 activation ↑ electron transport chain complexes I, II, III, and V
[24]	C2C12 Myoblasts	BBR 5 µM or HGSD-conditioned serum for 12 h Insulin 100 nM for 72 h	↑ Glucose consumption and uptake ↑ Insulin-stimulated GLUT4 translocation ↑ Insulin-stimulated p-AKT and p-IRS1 protein levels ↑ Insulin-stimulated PI3K association with p-IRS1 ↓ GR/GRα association with PI3K
[25]	C2C12 Myoblasts	BBR 1 μM, 5 μM, or 10 μM for 24 h	↑ Mitochondrial number ↑ p-AMPK ↑ Citrate synthase activity ↑ PGC-1α protein levels ↑ *mtTFA*, *NRF1*, *COXIV*, *CytC*, *PDK4*, *CPT1β*, *MCAD*, *LCAD* mRNA levels ↓ Basal oxygen consumption rate ↑ Maximal oxygen consumption rate
[26]	L6 myotubes	BBR 10 μM for 24 h Fructose 15 mM	↑ Glucose uptake ↓ Fructose-mediated elevation of IL-1β, IL-6 and TNF-α levels ↑ Fructose-mediated reduction in IL-10 levels ↓ ROS and MDA levels ↑ SOD, CAT, and GSH levels ↑ Nrf2 and HO-1 protein levels ↑ p-AKT and GLUT4 protein levels ↑ p-AMPK levels ↑ AMP/ATP ratio ↓ AMPD1 expression ↑ ADSL expression
[27]	C2C12 Myoblasts	BBR 5 μM, 25 μM, or 50 μM for 24 h PA 0.5 mM for 24 h	↓ Lipid droplet accumulation ↓ *TNFα* and *IL-6* mRNA levels ↓ FBXO32 and MuRF-1 protein levels ↑ FUNDC1 protein levels

Table Legend: ↑ = increase, ↓ = decrease, p- = phosphorylated. Only findings reported as statistically significant in the original manuscripts are included in this table.

**Table 2:** Effects of Berberine on Adipocytes (in vitro).

Source	Cell Type	Treatment	Findings
[28]	3T3-L1 Preadipocytes 3T3-L1 Adipocytes	BBR 5 μM or 10 μM or 20 μM for 6 days	↓ Preadipocyte differentiation ↓ ROS production ↓ Leptin and resistin protein levels ↑ *GPx* gene expression
[29]	3T3-L1 Adipocytes	BBR 1 μM, 5 μM, 10 μM, or 100 μM for 24 h Compound C 20 μM for 1 h	↓ Triglyceride content ↑ p-HSL (Ser565) protein levels ↑ *ATGL* mRNA levels ↑ p-AMPK levels
[30]	3T3-L1 Adipocytes	BBR 5 μM, 10 μM, 20 μM, or 40 μM for 24 h	↑ Glucose consumption and glucose transport ↓ *IL-6* and *TNF-α* mRNA levels ↓ *Leptin* mRNA levels ↑ *Visfatin* mRNA levels
[31]	3T3-L1 Preadipocytes	BBR 5 μM for 48 h	↑ ATP production and oxygen consumption ↑ UCP1, PRDM16, RBM4a protein levels ↓ Resistin, SRPK1 protein levels ↑ *miR-485* mRNA levels ↓ *miR-92a-5p* mRNA levels
[32]	Mouse primary Preadipocytes	BBR 5 μmol/L or 20 μmol/L for 10 days	↓Differentiation and proliferation ↓ Intracellular triglyceride content and lipid accumulation ↓ *PPARγ*, *C/EBPα*, and *aP2* mRNA levels ↓ *Gal-3* mRNA and protein expression ↑ let-7d expression
[33]	3T3-L1 Adipocytes	BBR 1 μM, 10 μM, or 100 μM for 3 days Dexamethasone 1 μM and insulin 1 μM for 3 days	↑ glucose usage ↑ adiponectin secretion ↓ fatty deposits of 3T3-L1 ↑ HIF3A, IRS-1, and GLUT4 protein levels
[34]	Porcine primary Preadipocytes	BBR 10 μM, 20 μM, 30 μM, or 40 μM for 24 or 48 h Compound C 10, 20 μM for 24 h	↑ Glycerol release ↓ Lipid content in fat droplets ↓ *Perilipin A* mRNA and protein levels ↑ p-ATGL protein levels ↑ p-AMPK protein levels ↑ *PGC-1α*, *TFAM*, *CPT-1* and *UCP2* mRNA levels
[35]	3T3-L1 Adipocytes	BBR 1 μM or 5 μM for 24 h TNF-α 1 ng/mL for 24 h	↑ Insulin-stimulated glucose uptake ↑ Glucose consumption ↑ SIRT1 protein expression ↑ *SIRT1*, *SIRT4*, and *SIRT6* mRNA expression ↑p-AMPK, p-ACC, and p-LKB1 expression ↑ p-AKT (Ser473) expression
[36]	3T3-L1 white Adipocytes HIB1b brown Adipocytes	BBR 5 μM for 4, 12 or 24 h AICAR 0.5 mM for 4 or 12 h Compound C 20 μM for 4 or 12 h	↑ p-AMPK expression ↑ Mitochondria number ↓ Lipid droplet size ↑ SIRT1 and UCP1 expression ↓ PPARγ acetylation ↑ NAD^+^/NADH ratio
[37]	Primary Porcine Intramuscular Preadipocytes	BBR 5 μM, 10 μM, or 20 μM for 48 h	↓ Intracellular triglyceride levels ↓ Adipocyte differentiation ↓ *PPAR-γ* and *Adiponectin* mRNA levels
[38]	3T3-L1 Adipocytes	BBR 20 μM, 40 μM, or 80 μM for 2 or 6 h TNF-α 15 ng/mL	↑ SIRT3 expression ↓ TNF-α-induced p-IKKα/β, p-IκBα and p65 levels ↓ p-JNK, p-p38, and p-ERK protein levels ↓ iNOS and COX-2 expression
[39]	3T3-L1 Adipocytes	BBR + evodiamine (BBE) 10 μM for 48 h	↓ Lipid accumulation ↓ Triglyceride content ↑ *Prdm16* and *Cidea* mRNA levels ↑ UCP1 and PGC-1a protein levels

Table Legend: ↑ = increase, ↓ = decrease, p- = phosphorylated. Only findings reported as statistically significant in the original manuscripts are included in this table.

**Table 3:** Effects of Berberine on Hepatocytes (in vitro).

Source	Cell Type	Treatment	Findings
[40]	HepG2	BBR 0, 1 μM, 5 μM, 10 μM, 15 μM, or 20 μM for 24 h Insulin 10^−6^, 10^−7^, 10^−8^ or 10^−9^ mol/L for 36 h	↑ Glucose uptake ↑ α7-nAChR protein expression ↓ AChE expression ↓ Insulin-induced IL-6 levels ↓ Insulin-induced p-IKKβ (Ser181) and NF-κB p65 protein levels
[41]	HepG2	BBR 10 μM for 24 h PA 0.3 mM for 24h	↓ G6Pase, PEPCK, SREBP-1, and FAS protein levels ↓ ACCα protein levels ↑ p-ACCα/ACCα ratio ↓ HNF4α and miR-122 expression
[42]	MIHA HepG2	BBR 10 μM, 20 μM, or 100 μM for 24 h	↑ *LDLR* mRNA levels ↑ miR-373 expression ↑ EGR1 expression ↓ AKT1, mTOR, and RICTOR protein expression
[43]	Primary Mouse Hepatocytes	BBR 5 μM for 2, 6 or 12 h AICAR 0.1 mM for 12 h Compound C 10 μM for 2 h Glucagon 10 nM for 2 h	↑ Glucose uptake ↓ Glucose production ↓ Oxygen consumption rate ↑ p-AMPK expression ↑ GLUT1 expression in plasma membrane ↓ SIRT3 expression ↑ Lactate output ↑ Intracellular AMP levels ↓ Intracellular cAMP levels ↓ Glucagon-induced gluconeogenesis ↓ PEPCK1 expression
[44]	Mouse AML12 Hepatocytes	BBR 1 μM, 5 μM, or 20 μM for 24 h Glucose 17. 5mM for 24 h	↑ GK expression ↑ Glycogen content
[45]	HepG2	BBR 20 μM for 24 h 25 mM glucose and 200 μM palmitic acid for 24 h	↓ palmitic acid-induced hepatic triglyceride levels ↓ SCD1 protein levels ↑ p-AMPK protein levels
[46]	HepG2	BBR 0.1 μM, 0.5 μM, or 2.5μ M for 24 h Oleic acid 0.6 mM for 24 h	↑ Glucose consumption ↓ Gluconeogenesis ↑ p-AMPKα1 protein levels ↑ p-ACC and p-Raptor protein levels ↓ *ChREBP* and *FAS* mRNA levels ↓ Oleic acid-induced lipid accumulation
[47]	HepG2	BBR 5 μg/mL, 10 μg/mL, or 20 μg/mL Insulin 10^−6^ mol/L for 36 h	↑ Glucose uptake and consumption ↓ TNF-*α*, IL-1*β*, IL-6, and IL-8 levels ↑ IL-10 levels ↑ PPM1B expression ↑ PPAR*γ*, LRP1, GLUT4, IRS-1, IRS-2, PI3K, and AKT expression ↓ *NF-κB p65* and *JNK* mRNA levels ↓p-IKK*β* Ser181/total IKK*β* ratio
[48]	Primary Mouse Hepatocytes	BBR 1 μM, 2 μM, or 4 μM for 24 h Oleic acid 0.8 mmol/L for 24 h	↓ Oleic acid-induced lipid droplets ↓ Oleic acid-induced triglyceride content ↓ ATP production ↑ p-AMPK and p-ACC levels
[49]	Primary Mouse Hepatocytes	BBR 10 μM for 15 min or 5 h TNF-α 10ng/mL for 15 min or 5 h Compound C 1 μM for 15 min Wortmannin 10 μM for 15 min Trametinib 10 μM for 15 min Insulin 10 nM for 5 min	↑ Insulin-stimulated p-AKT expression ↓ Insulin-stimulated p-IRS1 (Ser 612) ↓ p-ERK1/2
[50]	HepG2	BBR 10 μM for 24 h PA 0.3 mM for 24 h	↑ SIRT1 and Opa1 protein expression ↑ MMP ↑ p-AKT expression and glucose consumption
[51]	Primary Mouse Hepatocytes	BBR 20 μM for 16 h Cholesterol 30 μg/mL for 16 h	↓ Cholesterol-induced hepatocyte apoptosis ↑ Cell viability and proliferation ↓ ROS generation ↓ 4-HNE levels, NADPH oxidase activity, and iNOS expression ↓ Keap1 expression ↑ Nrf2 and HO-1 expression
[52]	Primary rat Hepatocytes	BBR 5 μM or 10 μM for 1.5 or 2 h Glucagon 10 nM for 4 h	↓ Basal and glucagon-stimulated gluconeogenesis ↓ G6Pase and PEPCK expression ↑ NO, cGMP, and PKG expression ↓ p-CREB expression ↑ p-AKT expression
[53]	HepG2	BBR 1.25 μM, 2.5 μM, or 5 μM for 18 h Glucosamine 20 mM for 18 h	↑ Glucose uptake ↓ ROS levels ↑ Bmal1 and Clock expression
[54]	HepG2	BBR 5 μM, 15 μM, 25 μM, 50 μM, 100 μM for 12 h Glucosamine Hydrochloride 18 mM for 18 h	↑ *FGF21* mRNA and protein expression and promoter activity ↑ p-AKT and p-GSK3β expression ↑ *PPARγ* mRNA and protein expression ↑ *GLUT2* mRNA and protein expression
[55]	HepG2 BRL-3A	BBR 2.5 μM, 5 μM, 10 μM for 48 h Fructose 20 mM for 48 h	↓ Fructose-induced IL-6 and TNF-α levels ↑ IL-10 levels ↓ ROS fluorescence and MDA ↑ SOD and GSH levels ↑ Nrf2, HO-1 and p-AMPK expression ↑ AMP/ATP ratio ↑ *ADK* mRNA and protein levels
[56]	HepG2 cells	BBR 5 μM for 24 h Oleic Acid 0.2 mM for 24 h	↑ Glucose uptake and consumption ↓ *AKR1B10* mRNA and protein levels ↑ CPT-1 and GK expression ↓ PPARγ expression ↑ p-GSK3β/GSK3β ratio
[57]	HepG2	BBR 20 μM for 24 h FFA 0.4 mM for 24 h	↑ Glucose uptake and consumption ↑ *PPARα*, *PPARγ* and *GLUT4* mRNA levels ↓ *GLUT1* mRNA levels
[58]	HepG2	BBR 5 μmol/L, 10 μmol/L, or 20 μmol/L for 24 h PA 200 μmol/L for 24 h	↓ Triglyceride and total cholesterol levels ↓ Lipid droplet size ↑ PI3K/Akt signaling ↓ STING pathway activation
[59]	HepG2	BBR 1 μM for 24 h MET 2 mM for 24 h Compound C 10 μM for 1 h before treatment PA 100 μM for 24 h Oleic Acid 200 μM for 24 h	↓ Intracellular lipid accumulation ↑ p-AMPK expression ↓ SREBP1 and FASN expression

Table Legend: ↑ = increase, ↓ = decrease, p- = phosphorylated. Only findings reported as statistically significant in the original manuscripts are included in this table.

**Table 4:** Effects of Berberine on Healthy Animal Models.

Source	Animal Type	Treatment	Findings
[60]	Male Wistar rats	BBR Orally 100 mg/kg/day for 6 months	↓ Aging-related learning and memory deficits ↓ Age-related increases in fasting blood glucose, insulin, and triglyceride levels ↑ Insulin-induced GLUT4 translocation ↓ Aging-induced muscle tissue deterioration ↓ ROS levels ↑ ATP production in skeletal muscle ↑ p-AMPK, SIRT1, and PGC-1α protein levels

Table Legend: ↑ = increase, ↓ = decrease, p- = phosphorylated. Only findings reported as statistically significant in the original manuscripts are included in this table.

**Table 5:** Effects of Berberine on HFD-Insulin Resistant Animals.

Source	Animal Type	Treatment	Findings
[61]	Golden Hamsters	BBR Orally 50 or 100 mg/kg/day for 6 weeks HFD (32% safflower oil, 33.1% casein, 17.6% sucrose for 8 weeks	↓ Body and liver weight ↓ Blood glucose levels ↓ Total cholesterol, Triglyceride, Free fatty acids, and LDL-cholesterol levels ↓ MDA plasma levels ↓ Thiobarbituric acid-reactive substance and 8-isoprostane levels ↑ GLUT4 expression in muscle ↑ *LDLR* mRNA levels in liver
[62]	Sprague–Dawley male rats	BBR Orally 200 mg/kg for a single dose HFD (51% fat) for 6 weeks	↓ Hepatic triglyceride levels ↓ ALT and AST ↑ *CPT-1α*, *MTTP*, and *GCK* mRNA levels ↑ CPT-1α, MTTP, and GCK protein levels
[63]	Male C57BL/6J mice	BBR Orally 100 mg/kg/day for 4 weeks HFD (60%)	↓ Plasma glucose levels ↓ p-JNK expression ↓ *IL-1β* and *TNFα* mRNA levels ↓ Hepatic steatosis ↓ *CPT1a* mRNA expression ↓ M1 Macrophage percentage
[64]	Male C57BL/6J mice	BBR Orally 150 mg/kg/day for 8 weeks HFD (60%)	↓ HFD-induced body weight gain ↓ Hepatic and serum triglyceride levels ↓ Hepatic liver droplet size ↓ Fas expression ↑ CYP4A10 expression
[65]	Male C57BL/6 mice Male ob/ob mice	BBR Orally 75 and 150 mg/kg/day for 3 weeks HFD (40%)	↑ Insulin sensitivity and glucose tolerance ↓ Fasting insulin serum levels ↓ Adipose tissue mass ↓ CD11c expression ↓ Caspase-1 expression
[66]	Male Wistar rats	BBR Orally 200 mg/kg/day for 8 weeks HFD (58% fat) for 28 weeks	↓ Fasting blood glucose ↓ Fasting insulin levels ↓ LDL-c and blood triglyceride levels ↓ HFD-induced liver weight gain ↓ Plasma LPS levels ↓ Hepatic *TLR4* and *TNF-α* mRNA ↑ *IR* and *IRS-1* mRNA levels Altered gut microbiome
[67]	Male C57BL/6J mice	BBR Orally 75 or 150 mg/kg/day for 4 weeks HFD (40%)	↑ Glucose tolerance ↓ Insulin resistance ↓ HFD-induced weight gain and fat accumulation ↓ FFA levels ↓ deposition of extracellular matrix in WAT ↓ HFD-induced expression of collagen type I, type III, and type VI ↑ p-AMPK and p-ACC protein levels ↓ HFD-induced TGF-β1 levels ↓ TNFα and iNOS protein levels ↑ Arg1 and Ym1 protein levels ↓ Macrophages infiltration
[68]	ICR male mice	BBR Orally 100 mg/kg/day for 10 weeks HFD (22.2% shortening)	↓ Fasting blood glucose ↓ Non-esterified fatty acid levels ↓ Endogenous glucose production ↓ *PEPCK* and *G-6-Pase* mRNA and protein levels ↓ hepatic triacylglycerol accumulation ↓ HFD-induced expression of Facd and Kct ↓ HFD-induced protein expression of MPC1 ↓ *SIRT3* gene expression Restrained mitochondrial pyruvate carboxylation
[69]	Male C57BL/6 mice	BBR Orally 100, 200, and 300 mg/kg/day for 8 weeks HFD (60%)	↓ Body weight ↓ Fasting blood glucose levels ↑ Glucose clearance rate and insulin sensitivity ↓ Adipose tissue fibrosis ↓ Collagen I transcription and fibronectin synthesis ↓ *α*-SMA and PDGFR-*α* expression ↓ HIF-1*α and LOX* expression
[70]	Male C57BL/6J mice	BBR Orally 100 mg/kg/day for 16 weeks HFD (60%)	↓ Fat content to body mass ratio ↓ Leptin and insulin serum levels ↓ Lipid droplet accumulation in liver and liver size ↑ UCP1 expression ↑ Oxygen consumption ↓ IL-6, IL-1β, TNF-α, and MCP1 expression ↑ IL-10 expression ↓ CD11b-positive and F4/80-positive cells ↑ *ARG1*, *MRC1*, and *CHIL3* gene expression ↓ caspase-3, caspase-8, and Bax expression in M2 macrophages ↓ IRE1α expression
[71]	Male C57BL/6J mice	BBR Intraperitoneally 1.5 mg/kg/day for 6 weeks HFD (60%) for 14 weeks	↑ Glucose metabolism ↓ HFD-induced iWAT and perirenal WAT mass ↑ BAT activity ↑ Energy expenditure ↑ O_2_ consumption and CO_2_ production ↓ Plasma insulin levels ↑ *Ucp1*, *Prdm16*, *Elovl3*, *Ppargc1a*, and *Ppargc1b* mRNA levels
[45]	Male C57BL/6N mice	BBR Orally 300 mg/kg/day for 4 weeks HFD (39.9%)	↓ HFD-induced hepatic steatosis ↓ Hepatic triglyceride accumulation ↓ SCD1 levels ↓ SREBP-1 and FAS levels ↑ Glucose tolerance ↓ Insulin resistance
[72]	Male Sprague–Dawley rats	BBR Orally 200 mg/kg/day for 4 weeks HFD (sucrose 20%, lard 15%, cholesterol 1.2%) + Streptozotocin injections for one week	↓ FBG, FINS, and IRI ↓ Total cholesterol LDL cholesterol levels ↑ ISI and HDL-C ↓ CORT levels in urine and serum ↓ ACTH plasma levels ↓ Hypothalamus Orexin-A, OX2R, and the CRH protein content ↑ GLUT4 protein and mRNA levels
[32]	Male C57BL/6 J mice	BBR Orally 100 mg/kg/day for 8 weeks HFD for 8 weeks	↓ Body weight ↓ Serum lipids, glucose, and insulin levels ↓ Gal-3 protein expression ↓ Adipocyte differentiation and proliferation ↑ Let-7d levels
[73]	Male C57BL/6J mice	BBR Intraperitoneally 1.5 mg/kg/day for 4 weeks HFD	↓ HFD-induced WAT weight ↓ Plasma triglyceride and fasting blood glucose levels ↓ IL-6 and MCP-1 levels ↑ IL-4, CD206, and ARG1 levels ↓ Macrophages infiltration ↓ CD68
[35]	Male C57BL/6J mice	BBR Orally 25 or 50 mg/kg/day for 2 weeks HFD (60%)	↓ Body weight and eWAT weight ↑ Glucose tolerance ↑ Insulin sensitivity ↓LDL-C serum levels ↓ Liver weight and triglyceride content ↓ ALT and AST levels ↑p-AMPK, p-ACC, p-AKT (Ser 473), and p-LKB1 expression ↓ MCP-1, IL-6, and TNF-α serum levels ↓ p-JNK and p-IKKβ expression ↓ PGC-1α acetylation
[36]	Male C57BL/6J mice	BBR Orally 25 or 100 mg/kg/day for 6 weeks HFD (60%)	↓ Body weight ↓ Blood glucose concentrations ↓ total cholesterol, triglyceride, and LDL-C levels ↑ Energy expenditure, VO_2_, and VCO_2_ ↓ I-WAT/body weight ratio ↑ BAT/body weight ratio ↓ Lipid droplets in BAT and iWAT ↑ UCP1 expression
[38]	Male C57BL/6 J mice	BBR Orally 200 mg/kg/day for 20 weeks HFD	↓ Body weight, eWAT, iWAT, BAT, and liver weight ↓ Fasting blood glucose levels ↑ Insulin sensitivity ↓ Total cholesterol, triglyceride, LDL-C, and NEFA serum levels ↑ HDL-C serum levels ↓ eWAT size ↓ *NOS*, *COX2*, *TNF-α, IL-1β* and *IFN-γ* mRNA levels ↓ *MCP-1*, *MIP-1α*, *Ccl5*, *Ccl11*, *Cx3cl1* and *Cxcl10* mRNA levels ↓ TNF-α-induced chemotaxis ↑ *CD206* and *CD301* mRNA levels ↓ Hepatic fibrosis and lipid accumulation ↓ AST and ALT levels ↑ SIRT3 expression
[74]	Male C57BL/6N mice	BBR Orally 300 mg/kg/day for 4 weeks HFD (60%) for 4 weeks	↓ Hepatic lipid accumulation ↓ HFD-induced body weight gain ↓ FFA, triglyceride, and cholesterol serum levels ↓ HFD-induced liver weight gain ↓ HFD-induced hepatic hypoxia ↓ HIF-2α expression ↓ ceramide levels ↑ Glycogen synthesis ↑ p-AKT, p-GSK3*β*, and p-PP2A
[53]	male C57BL/6 mice	BBR Orally 200 mg/kg/day for 4 weeks HFD (60%)	↓ NAS scores ↓ Fasting blood glucose and insulin serum levels ↓ Triglyceride and LDL-c serum levels ↓ ALT and AST serum levels ↓ ROS levels ↑ Bmal1 and Clock expression
[75]	Male C57BL/6J mice	BBR Orally 200 mg/kg/day for 4 weeks HFD (60%)	↓ HFD-induced body weight gain ↑ Glucose tolerance and insulin sensitivity ↓ Abnormal fasting blood glucose ↓ Triglyceride, total cholesterol, and LDL-c serum levels ↓ Triglyceride and cholesterol liver content ↓ HFD-induced liver weight gain and lipid accumulation ↓ AST and ALT levels ↑ SIRT1 transcription ↓ CPT1A protein levels
[55]	Male Sprague–Dawley Rats	BBR Orally 100 mg/kg/day for 10 weeks 30% Fructose in drinking water	↓ Blood glucose levels ↑ Glucose tolerance and insulin sensitivity ↓ ALT and AST serum levels ↓ Liver necrosis ↓ IL-6 and TNF-α serum levels ↑ IL-10 serum levels ↓ ROS and MDA levels ↑ SOD and GSH expression ↑ p-AMPK, Nrf2, HO-1 and ADK expression
[56]	C57BL/6 mice	BBR Orally 50 mg/kg/day for 4 weeks HFD	↓ Body weight ↓ Triglyceride levels in liver and serum ↓ Fasting glucose levels ↓ AKR1B10 protein levels ↓ PPARγ expression ↑ p-GSK3β/GSK3β ratio ↓ GLUT2, PEPCK and G6Pase expression
[27]	Four-week-old C57BL/6 mice	BBR Orally 100 mg/kg/day for 8 weeks HFD	↓ Body weight, body fat rate, and adipocyte size ↑ Glucose tolerance ↓ TNFα and IL-6 levels ↓ Lipid droplet deposits in skeletal muscle ↓ FBXO32 and MuRF-1 protein levels ↑ *LC3B* mRNA levels ↑ FUNDC1 protein levels
[58]	male C57BL/6 J mice	BBR Orally 50, 100, and 200 mg/kg/day for 4 weeks HFD	↓ Weight gain ↓ ALT and AST ↓ Liver inflammation and lipid accumulation ↓ Triglyceride and total cholesterol levels ↑ Glucose tolerance ↓ WAT size ↓ Fas, Scd1, and Nr1h3 expression ↑ Cpt1α expression ↑ p-PI3K and p-Akt expression ↓ p-TBK1/TBK1 and p-IRF3/IRF3 ratios
[76]	Male C57BL/6J mice	BBR Orally 150 or 300 mg/kg/day for 4 weeks HFD (60%)	↓ HFD-induced lipid droplet size ↓ Hepatic triglyceride content ↓ *Cidea* and *Plin4* mRNA levels
[77]	Male Syrian golden hamsters	HTD1801 Orally 100 or 200 mg/kg for 6 weeks HFD (1.25% cholesterol and 20% grease) for 8 weeks	↓ ALT and AST levels ↓ LDL-c and total cholesterol levels ↓ Hepatic fibrosis, steatosis, and inflammation
[59]	Male C57BL/6J mice	BBR Orally 100 mg/kg/day for 12 weeks MET 200 mg/kg/day for 12 weeks HFD (60%)	↓ hepatic-to-body mass ratio and visceral fat mass ↓ Fasting glucose levels and HOMA-IR ↓ Liver size ↓ Hepatic triglyceride and total cholesterol levels ↑ HDL-c levels ↓ LDL-c levels ↓ NAS ↓ ALT and AST levels ↓ TNF-α, IL-1β, and IL-6 levels ↑ p-AMPK expression
[78]	Female C57BL/6J mice	BBR Intravenously 10 μM for 10 weeks Nano-BBR Intravenously 10 μM for 10 weeks HFD (60%)	↓ Weight gain in nano-BBR group ↑ PRDM16 and CIDEA expression

Table Legend: ↑ = increase, ↓ = decrease, p- = phosphorylated. Only findings reported as statistically significant in the original manuscripts are included in this table.

**Table 6:** Effects of Berberine on Insulin-Resistant Genotypic and Phenotypic Animal Models.

Source	Animal Type	Treatment	Findings
[79]	Db/db male mice	BBR Orally 200 mg/kg/day for 5 weeks	↓ Diabetes-induced weight gain ↓ Insulin resistance ↓ Hepatic triglyceride levels ↓ Hepatic lipid accumulation ↓ ALT and AST levels ↓ Total cholesterol and LDL levels Inhibition of hepatic lipoperoxide accumulation ↓ SREBP-1c, CHREBP, FAS, and C/EBPβ ↓ *CYP2E1* and *CYP4A10* mRNA levels ↑ *Catalase* gene expression
[44]	Male C57BL/KsJ-db/db mice	BBR Orally 210 mg/kg/day for 4 weeks	↓ Fasting blood glucose, HbA1c, and glucagon levels ↑ Serum insulin ↑ Liver glycogen content ↓ Glucose-related metabolites ↑ GK expression ↑ *HK4* and *HK2* mRNA expression ↑ GLUT2 expression
[80]	Ob/ob mice Male C57BL/6J mice	BBR Orally 5 mg/kg/day for 3 weeks STZ Intravenously 100 mg/kg/day for 1 day	↓ Fed blood glucose and fasting plasma insulin in ob/ob mice ↓ Fasting blood glucose in STZ-induced diabetic mice ↑ Glucose tolerance and insulin sensitivity ↓ *Pepck*, *G6pase*, and *PGC1α* mRNA expression in liver ↓ Glucagon-stimulated blood glucose ↓ p-CREB expression ↓ Glucagon-stimulated cAMP expression
[24]	Male Wistar rats ICR mice	HFD/STZ rats: BBR 100 mg/kg/day or HGSD 25 or 100 mg/kg/day for 4 weeks HFD (22% fat and 48% carbohydrates) DEX mice: BBR or HGSD 150 mg/kg/day; DEX 2 mg/kg i.p. for 7 days	↓ FBG levels ↓ AUC for IPGTT ↑ Insulin-stimulated GLUT4 translocation ↑ Insulin-stimulated p-AKT protein levels ↑ Insulin-stimulated association of PI3K and p-IRS1 ↓ Association of PI3K and GR or GRα
[25]	Db/db male mice	BBR Orally 5mg/kg/day for 4 weeks	↓ Diabetes-induced weight gain ↓ Fasting blood glucose ↓ Fasting insulin levels ↓ FFA, triglyceride, and cholesterol serum levels ↓ Triglyceride content of muscle tissue ↑ Mitochondrial number ↑ Citrate synthase activity ↑ PGC-1α, ERRα, NRF-1, and mtTFA expression ↑ COXII, COXIV, and CytC and CPT1β expression ↑ p-AMPK protein levels
[81]	Male C57BL/6J wild-type mice and male leptin receptor- mutated (C57BL/KsJ-db/db) mice	BBR Orally 210 mg/kg/day for 28 days	↓ Hyperglycemia ↓ LDL-c and blood triglyceride levels
[82]	Male Sprague–Dawley rats Ppif+/+/Ppif−/− mice	BBR Orally 10mg/kg during intralipid-induced insulin resistance/clamp protocol Intralipid infusion for 3 h	↑ GIR at steady state ↓ Intralipid-induced mitochondrial swelling ↓ CypD protein expression
[83]	C57BL/6 mice	BBR Orally 75, 150, and 300 mg/kg/day for 3 days Tunicamycin 1 μg/g/day for 1 day	↓ Acute liver injury ↑ *CD36* mRNA levels ↓ FGF21 expression ↑ p-AKT expression ↓ *CHOP* and *GRP78* mRNA levels ↓ ATF6 expression
[50]	Male C57BL/6J mice Male Db/db mice	BBR Orally 160 mg/kg/day for 4 weeks HFD (60%)	↑ Glucose tolerance and insulin sensitivity ↓ Triglyceride and LDL-C levels ↓ Lipid accumulation ↓ *PEPCK* and *G6Pase* mRNA expression ↑ SIRT1 and Opa1 protein expression ↓ Drp1 protein expression
[52]	Spontaneous diabetic rats	BBR Orally 66.7 mg/kg/day for 6 weeks	↓ Hepatic glucose production ↓ G6Pase and PEPCK expression ↑ p-AKT expression ↑ NO and cGMP levels ↑ PKG signaling ↓ p-CREB expression
[54]	Male C57BL/6J mice Male C57BL/6J FGF21 knockout mice	BBR Orally 200 mg/kg/day for 6 weeks HFD (60%) + STZ intraperitoneally injection	↓ Body weight ↑ *FGF21* mRNA and protein expression ↓ Fasting blood glucose and serum insulin levels ↑ Insulin sensitivity and glucose tolerance ↑ p-AKT and p-AMPK expression ↑ PPARγ, FGF21, and GLUT2 expression ↑ Energy expenditure ↓ Liver lipid accumulation ↓ ALT, AST, and ALP levels ↑ Islet size of beta cells ↓ *IL-1β* mRNA levels
[84]	Male ob/ob mice Male C57BL/6 J mice	BBR Intraperitoneally 5 and 10 mg/kg/day for 4 weeks HFD	↓ Body weight, fat mass, and fat percentage ↓ eWAT and sWAT adipocyte size ↓ Hepatic lipid ectopic deposition ↓ Insulin and glucose plasma levels ↓ Total cholesterol, triglyceride, and free fatty acid levels ↑ Capillary number in adipose tissues ↑ CD31 and Ki67 expression ↑ VEGFA and VEGFR2 expression ↑ Mitochondria number ↑ PPAR-γ and UCP-1 expression ↑ *GPx3* gene expression

Table Legend: ↑ = increase, ↓ = decrease, p- = phosphorylated. Only findings reported as statistically significant in the original manuscripts are included in this table.

**Table 7:** Effects of Berberine in Humans (Clinical Studies).

Source	Participants	Treatment	Findings
[62]	Humans (males and females) (39–61 years old) (BMI = 24–30 kg/m^2^) *n* = 184	BBR Orally 500 mg capsule 30 min before mealtime 3 times daily (1500 mg daily) for a 16-week period	↓ Weight, BMI, and Waist circumference ↓ Hepatic fat content ↓ Total cholesterol, triglycerides, and ApoB levels ↑ ApoA levels ↓ ALT and AST levels ↓ Fasting blood glucose and HOMA-IR
[85]	Humans (males and females) (Average age of 40 years old) *n* = 80	BBR Orally 500 mg 30 min before meals, 3 times daily (1500 mg daily) for 16 weeks	↓ Weight, BMI, and waist circumference ↓ Hepatic fat content ↓ ALT, AST, γ-GT ↓ OGTT blood glucose levels ↓ Fatty acid, Ceramide, sphingomyelin, lysophosphatidylinositol, phosphatidylinositol, and lysophosphatidylcholine levels
[86]	Humans (males and females) (22–70 years old) (BMI = 25–40 kg/m^2^) *n* = 50	BBR ursodeoxycholate Orally 500 mg or 1000 mg or 2000 mg daily for 28 days	↓ Triglyceride, total cholesterol, LDL cholesterol, and Non-HDL cholesterol levels
[87]	Females (20–35 years old) (BMI = 20–30 kg/m^2^) *n* = 12	BBR Phytosome Orally 550 mg capsule twice daily before lunch and dinner for 60 days	↓ HOMA-IR ↓ Glycemia ↓ Insulin levels ↓ VLDL and triglycerides ↓ Testosterone ↑ SHBG ↓ Acne symptoms ↓ BMI, hip circumference, waist circumference, waist/hip ratio, VAT, total mass, and fat mass ↓ CRP and TNFα
[88]	Humans (males and females) (26–75 years old) (BMI = 25–55kg/m^2^) *n* = 100	BBR Ursodeoxycholate Orally 500 mg or 1000 mg twice a day for 18 weeks	↓ Liver fat content ↓ HbA1c levels ↓ ALT and GGT levels ↓ LDL-c and triglyceride levels ↓ Body weight
[89]	Humans (males and females) (51–67 years old) (BMI = 26.73–33.59 kg/m^2^) *n* = 49	BBR Phospholipid/BBR Phytosome™ Orally 550 mg tablet twice daily before lunch and dinner for 60 days	↓ Glycemia ↓ Total cholesterol, triglyceride, and insulin levels ↓ ApoB and ALT levels ↓ Waist circumference, VAT, and fat mass
[90]	Humans (males and females) (18–55 years old) *n* = 34	HIMABERB (BBR) Orally 500 mg capsule 3 times daily for 12 weeks	↓ FPG, FI, h-OGTT, HOMA-IR, HbA1c levels ↓ ALP, AST, and ALT levels
[91]	Humans (males and females) (40–59 years old) (BMI = 25.8–39.9 kg/m^2^) *n* = 70	BBR Orally 1500 mg/day Two 250 mg capsules an hour before mealtime 3 times daily for a 12-week period	↓ Trunk fat ↓ ALT levels ↓ De Ritis ratio ↓ Total cholesterol levels
[92]	Humans (males and females) (37–43 years old) *n* = 50	BBR Orally 500 mg capsule three times a day for a 3-month period	↓ Visfatin and Apelin levels ↑ Omentin levels ↓ Body weight and BMI ↓ Body fat percentage and visceral fat percentage ↓ Diastolic and systolic blood pressure ↓ Waist-hip ratio
[77]	Humans (males and females) (26–75 years) *n* = 100	HTD1801 (BBR ursodeoxycholate) Orally 500 mg or 1000 mg twice daily for 18 weeks	↓ Liver fat content and body weight ↓ ALT levels ↑ MASH resolution index
[93]	Humans (males and females) (Mean age 54.3 [10.6]) (Mean BMI 25.5 kg/m^2^, HbA1c 8.2%) *n* = 113	HTD1801 (BBR ursodeoxycholate) Orally 500 mg or 1000 mg twice daily for a 12-week period	↓ HbA1c levels ↓ Fasting plasma glucose levels, HOMA-IR, fasting insulin levels ↓ Total cholesterol and LDL-C levels ↓ ALT and AST levels ↓ GGT levels
[94]	Humans (males and females) (Age not reported) *n* = 33	BBR or dhBBR Orally 500 mg three times a day for 15 days	↓ GGT, ALP, ALT, and AST levels ↓ 5-HT serum concentrations
[95]	Humans (males and females) (Average age of 41 years) *n* = 337	BBR hydrochloride Orally 500 mg twice daily for 6 months	↓ LDL-c and ApoB levels ↓ hs-CRP levels

Table Legend: ↑ = increase, ↓ = decrease, p- = phosphorylated. Only findings reported as statistically significant in the original manuscripts are included in this table.

## Data Availability

No new data were created or analyzed in this study. Data sharing is not applicable to this article.
